# LncGSAR Controls Ovarian Granulosa Cell Steroidogenesis via Sponging MiR-125b to Activate SCAP/SREBP Pathway

**DOI:** 10.3390/ijms232012132

**Published:** 2022-10-12

**Authors:** Yong Wang, Yunxia Guo, Chunhui Duan, Junjie Li, Shoukun Ji, Huihui Yan, Yueqin Liu, Yingjie Zhang

**Affiliations:** 1 Laboratory of Small Ruminant Genetics, Breeding and Reproduction, College of Animal Science and Technology, Hebei Agricultural University, Baoding 071000, China; 2 College of Life Science, Hebei Agricultural University, Baoding 071000, China

**Keywords:** lncGSAR, MiR-125b, ceRNA, granulosa cell, steroidogenesis

## Abstract

Long non-coding RNAs (lncRNAs) have been shown to play important roles in livestock fecundity, and many lncRNAs that affect follicular development and reproductive diseases have been identified in the ovary. However, only a few of them have been functionally annotated and mechanistically validated. In this study, we identified a new lncRNA (lncGSAR) and investigated its effects on the proliferation and steroidogenesis of ovine granulosa cells (GCs). High concentrations of glucose (add 33.6 mM glucose) caused high expression of lncGSAR in GCs by regulating its stability, and lncGSAR overexpression promoted GCs proliferation, estrogen secretion, and inhibited progesterone secretion, whereas interference with lncGASR had the opposite effect. Next, we found that the RNA molecules of lncGSAR act on MiR-125b as competitive endogenous RNA (ceRNA), and SREBP-cleavage-activating protein (SCAP) was verified as a target of MiR-125b. LncGASR overexpression increased the expression of SCAP, SREBP, and steroid hormone-related proteins, which can be attenuated by MiR-125b. Our results demonstrated that lncGSAR can act as a ceRNA to activate SCAP/SREBP signaling by sponging MiR-125b to regulate steroid hormone secretion in GCs. These findings provide new insights into the mechanisms of nutrient-regulated follicle development in ewes.

## 1. Introduction

High reproductive efficiency (i.e., litter size) is the core of the sheep breeding industry, and the productivity of a breeding ewe determines its commercial value. Normal proliferation and steroidogenesis of follicle granulosa cells (GCs) are crucial for ovarian follicular development, oocyte maturation, and subsequent embryonic development [1,2]. Therefore, the discovery of genetic regulatory factors involved in GCs function is of great significance for improving ovulation rates and ewe fertility.

Normal glucose metabolism in GCs is essential for oocyte development and maturation as well as the protection of GCs development [3,4]. However, chronically high glucose levels have deleterious effects on the structure and function of the ovary, especially oocytes and GCs during folliculogenesis [5]. These complex cellular and development processes depend on the precise spatiotemporal expression of regulatory factors. Our previous studies found that glucose dose had a direct effect on GCs proliferation, apoptosis, and steroid hormone secretion, and glycolytic metabolites were positively correlated with steroid hormone secretions [6,7]. This confirms the connection between GCs steroidogenesis and glucose availability, but the molecular mechanisms underlying these changes require further study.

Long non-coding RNAs (lncRNAs) are a class of non-coding RNAs that are longer than 200 nucleotides in length and no protein-coding potential, which are involved in numerous important biological processes [8]. In reproductive activities, lncRNAs played diverse roles in the regulation of follicular development [9], oocyte maturation [10], GCs differentiation [11], and reproductive diseases [12,13]. For example, NEAT1, a lncRNA, was demonstrated to facilitate ovarian GCs proliferation and inhibit apoptosis by regulating the IGF1 [14]. At the post-transcriptional level, lncRNA can serve as efficient microRNA (miRNA) sponges—termed competing endogenous RNAs (ceRNAs)—that interact with miRNA to regulate gene expression [15]. For example, FDNCR sponges miR-543-3p in GCs and prevents miR-543-3p from binding to the DCN 3′ UTR, resulting in DCN transactivation and TGF-b pathway inhibition and promotion of GCs apoptosis in Hu sheep [16]. In addition, lncRNA NORHA acts as a “sponge” that directly binds to the miR-183-96-182 cluster to induce apoptosis in porcine ovarian GCs [17]. Although functions of these lncRNAs have been partially characterized, most of their roles for steroid hormone secretion are still poorly understood.

In this study, to explore the role of lncRNAs in regulating glucose-stimulated ovarian GCs steroidogenesis, ovarian GCs cultured with different glucose doses (optimum glucose concentration (8.4 mM) and high glucose concentration (33.6 mM)) [6,7] were used for RNA sequencing (RNA-seq). Based on this result, we investigated the function of a novel lncRNA named granulosa cell steroidogenesis-associated RNA (GSAR). Moreover, the function and underlying regulatory mechanisms of lncGSAR were further explored, and our results elucidated that mechanism of lncGSAR served as a ceRNA regulating ovarian GCs proliferation and steroid hormone secretion. These results provide some clues to the lncRNAs mechanisms of regulation in nutrient-stimulated follicle development and ovulation.

## 2. Results

### 2.1. LncRNA-miRNA-mRNA Interaction Network

**Figure 1:**
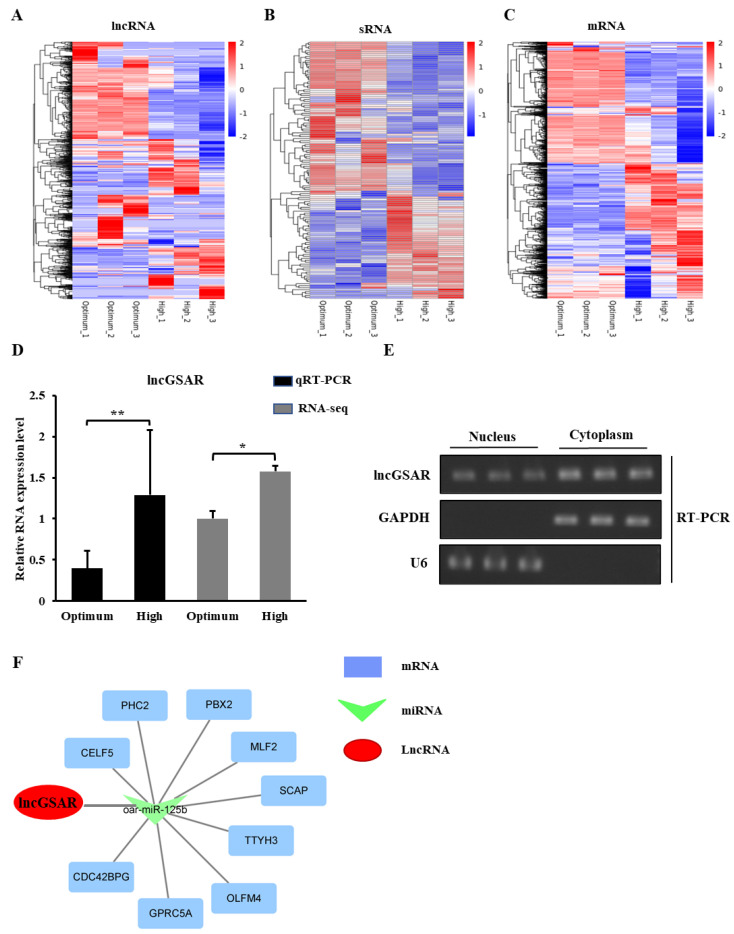
LncRNA-miRNA-mRNA interaction network. (**A**–**C**) Heatmap of long non-coding RNAs (lncRNAs) (**A**), microRNA (miRNA) (**B**), and mRNAs (**C**) showing hierarchical clustering of changed lncRNAs, miRNA, and mRNAs of granulosa cells (GCs) in different glucose treatment groups; up- and downregulated genes are colored in red and blue, respectively. (**D**) Quantitative real-time PCR (qRT-PCR) validations and RNA sequencing (RNA-seq) of lncGSAR in GCs from in different glucose treatment groups. (**E**) LncGSAR is localized in the cytoplasm and nucleus. GAPDH and U6 serve as cytoplasmic and nuclear localization controls, respectively. (**F**) LncRNA-miRNA-mRNA interaction network consists of one lncRNA (red circle), one miRNA (green arrow), and nine mRNAs (blue squares). Values represent means ± SEM for three individuals. * *p* < 0.05 and ** *p* < 0.01.

There are 461 (281 upregulated and 180 downregulated) differentially expressed lncRNAs (Figure 1A, Table S3), 94 (47 upregulated and 47 downregulated) differentially expressed miRNAs (Figure 1B, Table S4), and 796 (379 upregulated and 417 downregulated) differentially expressed genes (Figure 1C, Table S5) that were identified with a *p*-value < 0.05 as the cut-off. To determine the possible functional significance of observed changes in lncRNA levels in the optimum and high-glucose-induced GCs groups, a gene ontology (GO) term enrichment analysis was performed. We found the differentially expressed lncRNAs were found to be similar and significantly associated with steroid hormone stimulus in biological process term enrichment (Figure S1). Interestingly, the Kyoto Encyclopedia of Genes and Genomes (KEGG) enrichment analysis of lncRNA target genes also found the ovarian steroidogenesis pathway and steroid hormone biosynthesis pathway were both enriched in comparison group (Figure S2). These findings suggest that steroidogenesis may play critical roles in glucose-induced function of GCs.

Protein-coding RNAs (mRNAs) and lncRNAs, which share the common miRNA response elements (MREs), can both compete for binding to miRNAs and regulate each other. In this study, we constructed a putative lncRNA–miRNA–mRNA crosstalk network involved in GCs steroidogenesis. A novel differentially expressed lncRNA (the intergenic lncRNA TCONS_00219351 is located on chromosome chr26: 25174136-25228108, and its RNA sequence is shown in Figure S3) was served as a candidate. The expression of lncRNA TCONS_00219351 was significantly up-regulated in the high-glucose group compared with the optimal-glucose concentration group, and the analysis of qRT-PCR also verified this result (Figure 1D). Thus, we suspected that lncRNA TCONS_00219351 plays an important role in steroid hormone synthesis of GCs and hereinafter refer to this lncRNA as lncGSAR for convenience. We further investigated the subcellular localization of lncGSAR, and the RT-PCR result confirmed that it is an RNA molecule present in the cytoplasm and nucleus (Figure 1E). The lncGSAR-binding miRNA were predicted as candidates using RNAhybrid and miRanda software. A ceRNAs network (lncGSAR–miRNA–mRNA) with 1 miRNA and 10 mRNAs was constructed in GCs (Figure 1F, Table S6). Notably, SREBP-cleavage-activating protein (SCAP) gene from lncRNA–miRNA–gene network was a critical sensor of glutamine, glucose, and sterol levels [18]. SCAP regulates sterol production by activating the sterol regulatory element-binding proteins (SREBPs) signaling pathway, which plays an important role in the subsequent steroid hormone synthesis [19].

### 2.2. High Glucose Regulates lncGSAR Expression by Affecting Its Stability

**Figure 2:**
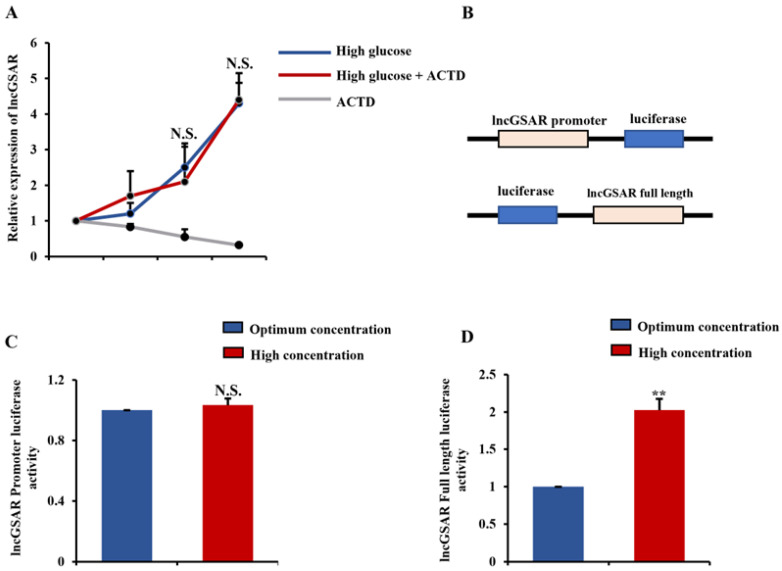
LncGSAR is transactivated upon glucose stimulation. (**A**) The GCs were treated with actinomycin D to explore the effect of glucose on the expression of lncGSAR. (**B**) Dual-luciferase reporter assay was conducted of lncGSAR promoter sequence and full-length sequence. (**C**) Dual-luciferase reporter for the lncGSAR promoter. (**D**) Detection of lncGSAR full-length luciferase activity. Values represent means ± SEM for three individuals. ** *p* < 0.01. N.S., not significant.

To validate the effect of different glucose on the expression of lncGSAR, we treated g GCs with high-concentration glucose with or without actinomycin D (ACTD), which is an RNA transcription inhibitor. As shown in Figure 2A, the lncGSAR level was increased after high-concentration glucose treatment; however, the enhanced lncGSAR fails to be rescued by ACTD, which suggests high-concentration glucose regulated lncGSAR expression dependent on enhancing its stability. To further explore the mechanism of glucose enhances lncGSAR stability, we cloned the full-length lncGSAR sequence after luciferase element of pMIR vector to obtain pMIR-luciferase lncGSAR and cloned the lncGSAR promoter before the luciferase element of Pgl3 vector to obtain Pgl3-lncGSAR promoter luciferase plasmids, respectively (Figure 2B). It is no surprise that the luciferase activity of Pgl3-lncGSAR promoter luciferase cannot be changed, yet the activity of pMIR-luciferase lncGSAR was increased under high-level glucose treatment (Figure 2C,D). Thus, these data indicate expression of lncGSAR was increased through reinforcing its stability under high-glucose stress.

### 2.3. LncGSAR Promotes Steroid Hormone Secretion and Proliferation of GCs

**Figure 3:**
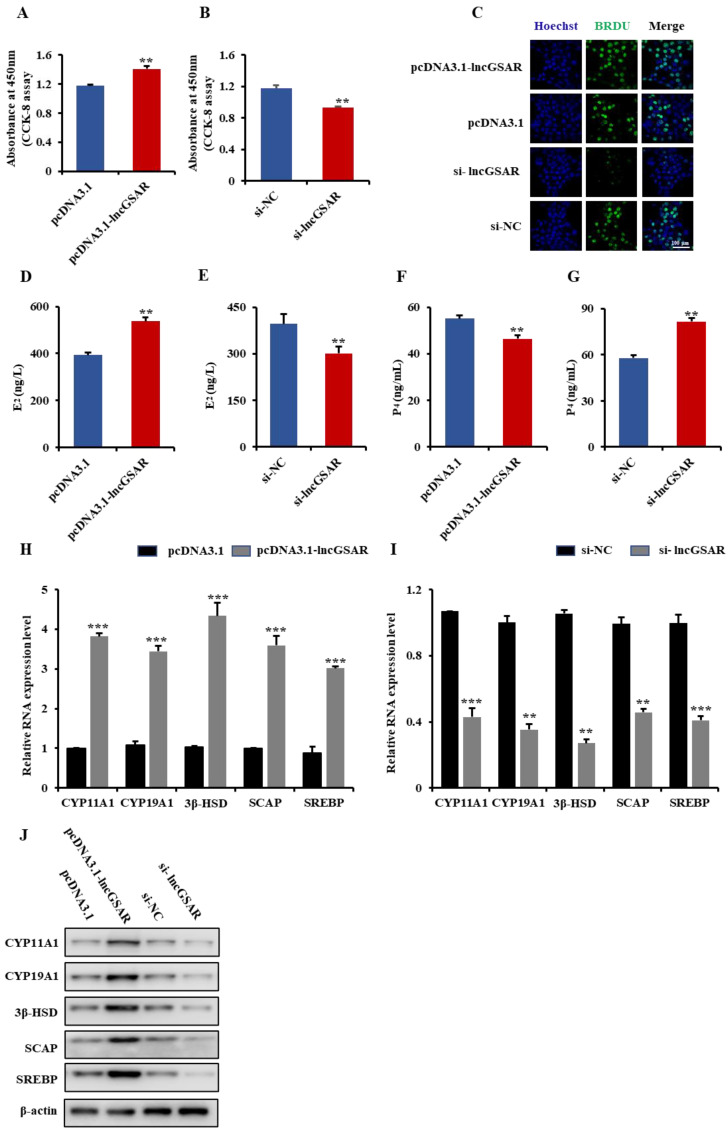
LncGSAR promotes GCs steroidogenesis and proliferation. (**A**,**B**) CCK-8 assay was performed to assess the effect of lncGSAR overexpression and knockdown on GCs proliferation. (**C**) The 5′-bromo-2′-deoxyuridine (BrdU) analysis after transfection of pcDNA3.1-lncGSAR and pcDNA3.1 empty plasmids, i.e., si-lncGSAR and si-NC, in proliferating GCs; scale bars are 100 µm. (**D**,**E**) Determination of estradiol (E_2_) concentrations in GCs transfected with pcDNA3.1-lncGSAR and pcDNA3.1 empty plasmids, i.e., si-lncGSAR and si-NC. (**F**,**G**) The progesterone (P_4_) concentrations in GCs after transfection of pcDNA3.1-lncGSAR and pcDNA3.1 empty plasmids or si-lncGSAR and si-NC. (**H**,**I**) The qRT-PCR of relative expression levels of steroidogenesis-related (CYP11A1, CYP19A1, and 3β-HSD) and sterol regulation-related (SCAP and SREBP) mRNAs in GCs transfected with pcDNA3.1-lncGSAR and in GCs transfected with si-lncGSAR. (**J**) Overexpression and knockdown of lncGSAR affected the expression of steroidogenesis-related proteins and sterol regulatory element proteins. Values represent means ± SEM for three individuals. ** *p* < 0.01; *** *p* < 0.001.

In order to assess the function of lncGSAR in GCs, lncGSAR overexpression vector (pcDNA3.1-lncGSAR) or lncGSAR small interfering RNA (si-lncGSAR) were constructed and transfected into GCs. The cell counting kit-8 (CCK-8) assay showed that overexpression of lncGSAR significantly increased GCs viability (Figure 3A). The 5′-bromo-2′-deoxyuridine (BrdU) detection also demonstrated that the proliferation rate of lncGSAR overexpression cells was significantly increased compared with that of the control (Figure 3C). Conversely, the opposite result was observed by lncGSAR interference (Figure 3B,C), indicating that lncGSAR can facilitate GCs proliferation.

To further investigate the role of lncGSAR in secretion of steroid hormones, we measured the concentrations of estradiol (E_2_) and progesterone (P_4_) in GCs by enzyme-linked immunosorbent assay (ELISA). The result revealed that overexpression of lncGSAR significantly increased concentrations of E_2_ (Figure 3D) but decreased concentrations of P_4_ (Figure 3F). In addition, we found that lncGSAR overexpression significantly upregulated expression levels of steroidogenesis-related (CYP11A1, CYP19A1, and 3β-HSD) and sterol regulation-related (SCAP and SREBP) mRNAs (Figure 3H). Western blot analysis also showed that overexpression of lncGSAR significantly improved the expression levels of steroidogenesis-related proteins and sterol regulatory element proteins (Figure 3J). On the contrary, lncGSAR interference inhibited E_2_ secretion (Figure 3E) but promoted P_4_ secretion (Figure 3G) as well as significantly downregulated mRNA and protein expression levels of steroidogenesis-related and sterol regulation-related genes (Figure 3I,J). These results suggest that lncGSAR might play a crucial role in the regulation of steroidogenesis in GCs.

### 2.4. LncGSAR Acts as a Molecular Sponge for MiR-125b

**Figure 4:**
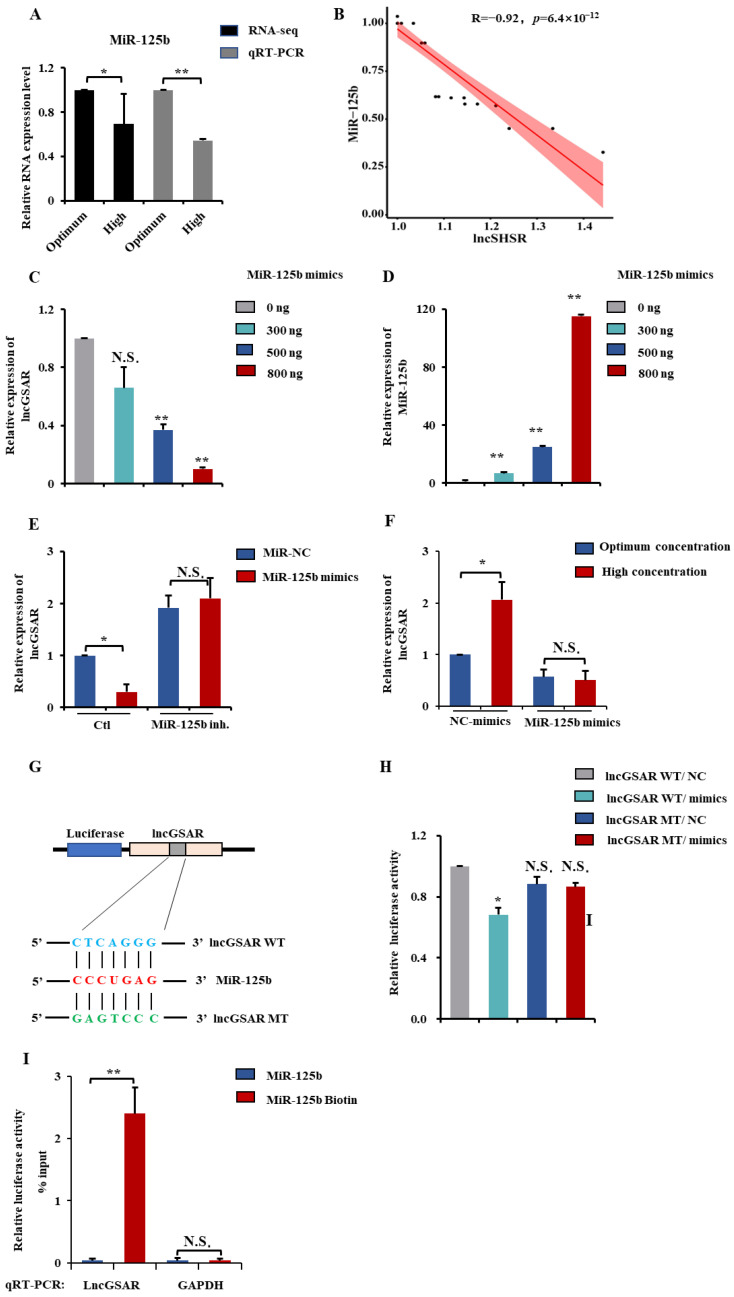
LncGSAR functions as a competing endogenous RNAs for MiR-125b. (**A**) The qRT-PCR validations and RNA-seq of MiR-125b in GCs from in different glucose treatment groups. (**B**) Pearson’s correlation was determined between lncGSAR and MiR-125b. (**C**,**D**) The expression levels of lncGSAR (**C**) and MiR-125b (**D**) in GCs after transfection of 0, 300, 500, or 800 ng of MiR-125b mimics. (**E**) Detection of lncGSAR expression levels after transfection of MiR-125b mimics or MiR-125b inhibitor. (**F**) Detection of lncGSAR expression levels after transfection of MiR-125b mimics in different glucose concentrations groups. (**G**) Schematic depicting the interactions of MiR-125b with wild-type lncGSAR (blue) and mutant lncGSAR (green). Red nucleotides indicate the seed sequence of MiR-125b. (**H**) The regulatory relationship between lncGSAR and MiR-125b was assessed using a dual-luciferase reporter gene assay. (**I**) The interaction of lncGSAR with MiR-125b was determined by RNA-RNA interaction pull-down assay. Values represent means ± SEM for three individuals. * *p* < 0.05; ** *p* < 0.01. N.S., not significant.

MiR-125b is the core component of lncRNA–miRNA–gene network and has been reported to play a critical role in reproductive hormone biosynthesis and follicle development [20]. In addition, MiR-125b regulated GCs apoptosis in the yak ovary by targeting BMPR1B [21]. In the present study, the lncRNA–miRNA–gene network suggests both the lncGSAR and MiR-125b exhibits the deeply potential to interact with each other (Figure 1F). Furthermore, the RNA-seq showed that the expression of MiR-125b was significantly downregulated in the high-glucose group, and the analysis of qRT-PCR also verified this result (Figure 4A). There was a significant negatively correlation between the MiR-125b expression and lncGSAR expression (Figure 4B).

To explore the regulatory relationship between lncGSAR and MiR-125b, granulosa cells were transfected with different doses of MiR-125b mimics (0, 300, 500, and 800 ng) for 48 h. We found that the expression levels of lncGSAR decreased with increasing doses of MiR-125b mimics (Figure 4D), while expression levels of MiR-125b gradually increased (Figure 4C). Next, we added MiR-125b inhibitor to the MiR-125b mimic group and MiR-NC group, respectively, and found that MiR-125b inhibitor could rescue MiR-125b-induced downregulation of lncGSAR expression level (Figure 4E). It is worth noting that addition of MiR-125b mimic reversed the upregulation of lncGSAR expression by high glucose so that the mRNA expression level of lncGSAR had no significant difference between the high-glucose group and the optimal-concentration group (*p* < 0.05) (Figure 4F). To further investigate the MiR-125b directly binding to lncGSAR, dual-luciferase reporter constructs containing the miRNA response element (MRE; wildtype (WT)) and mutant (MT) plasmid were co-transfected with MiR-125b mimics into GCs (Figure 4G). Luciferase activity of the lncGSAR-WT was dramatically decreased; nevertheless, MiR-125b failed to inhibit the lncGSAR-mut luciferase activity. (Figure 4H). Meanwhile, the RNA–RNA binding assay showed that MiR-125b directly contacts with lncGSAR and not the control RNA GAPDH (Figure 4I).

### 2.5. MiR-125b Suppresses GCs Proliferation and Steroidogenesis

**Figure 5:**
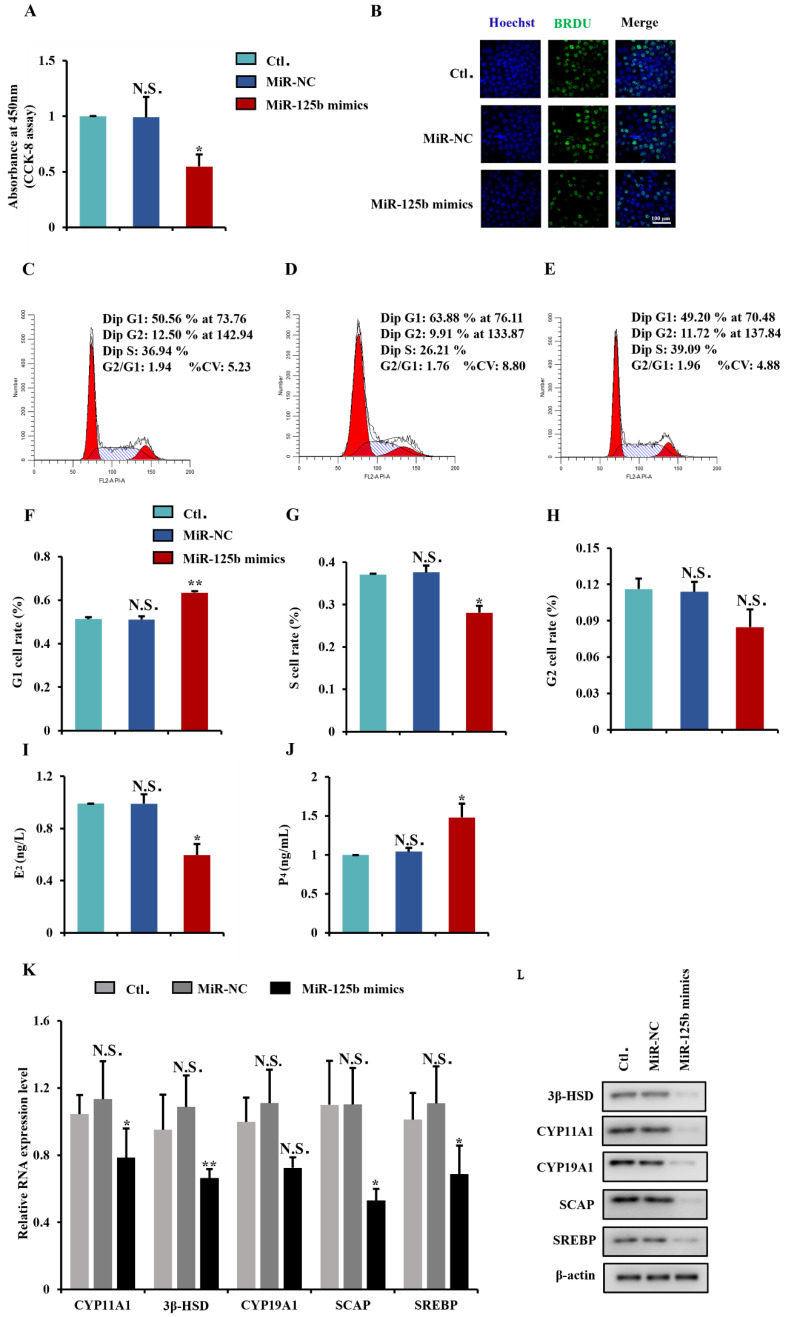
MiR-125b inhibit GCs steroidogenesis and proliferation. (**A**) The cell counting kit-8 (CCK-8) assay of GCs at 48 h after transfection of MiR-125b mimics and MiR-NC. (**B**) BrdU analysis was performed to assess the effect of MiR-125b overexpression on GCs proliferation. (**C**–**H**) Cell cycle analysis of GCs at 48 h after transfection of MiR-125b mimics plasmid. (**I**) Determination of E_2_ concentrations after 48 h of GCs transfected with MiR-125b mimics. (**J**) Determination of P_4_ concentrations after 48 h of GCs transfected with MiR-125b mimics. (**K**) The qRT-PCR of relative expression levels of steroidogenesis-related (CYP11A1, CYP19A1, and 3β-HSD) and sterol regulation-related (SCAP and SREBP) mRNAs in GCs transfected with MiR-125b mimics. (**L**) Overexpression of MiR-125b affected the expression of steroidogenesis-related proteins and sterol regulatory element proteins. Values represent means ± SEM for three individuals. * *p* < 0.05; ** *p* < 0.01. N.S., not significant.

To further explore the critical functions of MiR-125b in GCs, we transfected GCs with miRNA mimics or miRNA-negative control (MiR-NC) and then monitored the proliferation status of cells using CCK-8 assay, BrdU staining, and flow cytometry analysis. CCK-8 and BrdU staining demonstrated that the proliferation rate of MiR-125b-transfected GCs was significantly reduced compared with that of the MiR-NC-transfected cells (Figure 5A,B). Flow cytometry analysis of the cell cycle revealed that GCs transfected with the MiR-125b mimic could elevate the ratio of cells that progressed to the G1 phase and reduced the ratio of cells that progressed to the S phase (Figure 5C–H). After ELISA, we found MiR-125b overexpression significantly inhibited concentrations of E_2_ but significantly promoted production of P_4_ (Figure 5I,J). The expression levels of steroidogenesis- related (CYP11A1, CYP19A1, and 3β-HSD) and sterol regulation-related (SCAP and SREBP) mRNAs were downregulated in MiR-125b overexpression GCs compared to MiR-NC transfected GCs (Figure 5K). In addition, overexpression of MiR-125b reduced the expression level of steroidogenesis-related proteins and sterol regulatory element proteins (Figure 5J). Together, these results suggest that MiR-125b inhibits GC proliferation and steroidogenesis.

### 2.6. MiR-125b Regulates GCs Proliferation and Steroidogenesis by Targeting SCAP-SREBP Axis

**Figure 6:**
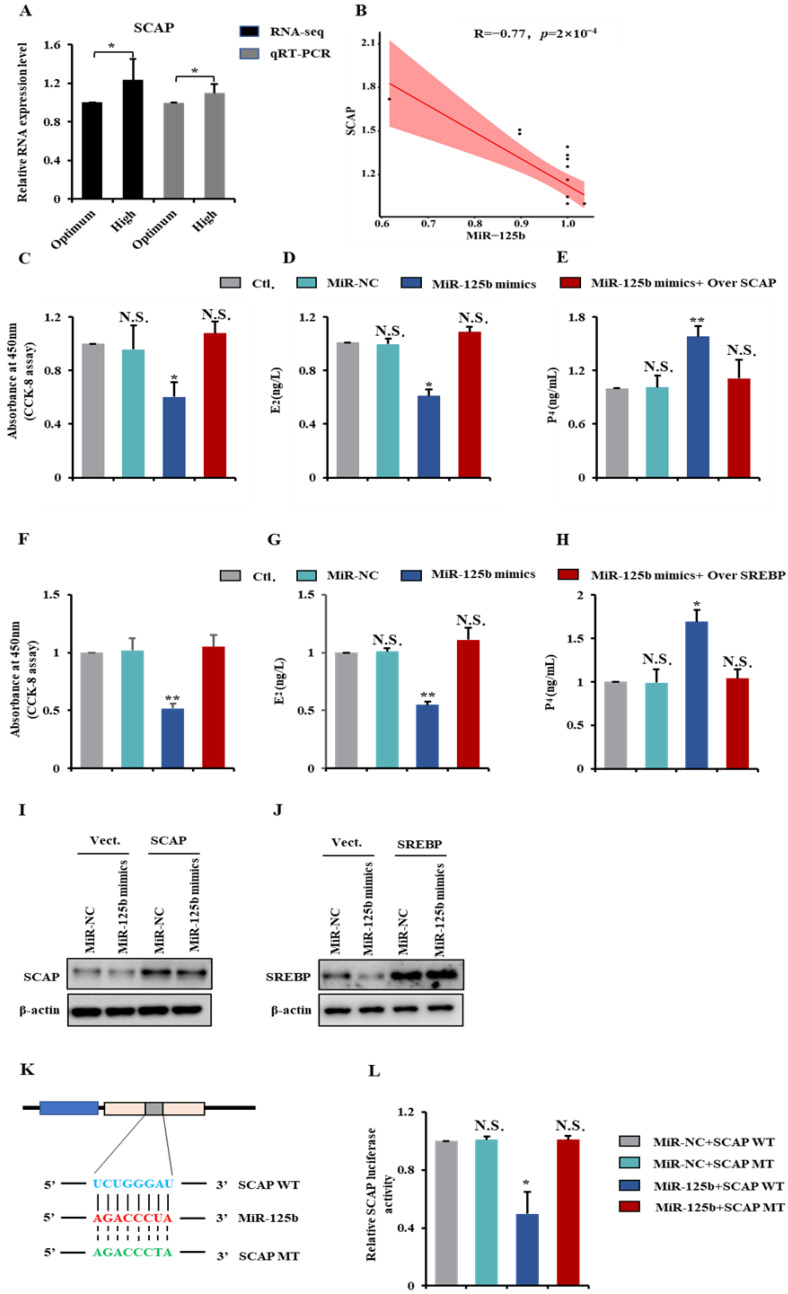
MiR-125b regulates GCs proliferation and steroidogenesis and by targeting SCAP-SREBP axis. (**A**) The qRT-PCR validations and RNA-seq of SCAP in GCs from in different glucose treatment groups. (**B**) Pearson’s correlation was determined between SCAP and MiR-125b. (**C**) MiR-125b or pcDNA3.1-lncGSAR and MiR-125 mimics were co-transfected into GCs for CCK-8 assay. (**D**,**E**) The concentrations of E_2_ and P_4_ determination in GCs transfected with MiR-125b mimics, i.e., pcDNA3.1-lncSCAP and MiR-125 mimics. (**F**) CCK-8 assay of GCs at 48 h after transfection of MiR-125b mimics plasmid or pcDNA3.1-lncSREBP and MiR-125 mimics. (**G**,**H**) The concentrations of E_2_ and P_4_ determination in GCs transfected with MiR-125b mimics, i.e., pcDNA3.1-lncSREBP and MiR-125 mimics. (**I**,**J**) Western blot detection of SCAP (**I**) and SREBP (**J**) in GCs after overexpression of MiR-125b. (**K**) Schematic depicting the interaction of MiR-125b with wild-type (blue) and mutant SCAP (green). Red nucleotides indicate the seed sequence of MiR-125b. (**L**) The regulatory relationship between MiR-125b and SCAP was assessed using a dual-luciferase reporter gene assay. * *p* < 0.05; ** *p* < 0.01. N.S., not significant.

Based on the above ceRNA (lncGSAR–MiR-125b–mRNA) network, several genes (e.g., SCAP and GPRC5A) were predicted to be MiR-125b target genes. SCAP, a transmembrane structural protein located on the endoplasmic reticulum and functioning as a sterol sensor, attracts our attention [22]. Thus, a SCAP was selected as a candidate target of the MiR-125b for further study. In the present study, the RNA-seq and qRT-PCR both showed that the expression of SCAP was significantly upregulated in the high-glucose group (Figure 6A). There was a significant negatively correlation between the MiR-125b expression and SCAP expression (Figure 6B). Meanwhile, the proliferation rate and E_2_ concentrations of MiR-125b transfected GCs were significantly downregulated (Figure 6C,D), while the P_4_ concentration was upregulated (Figure 6E). However, overexpression of SCAP alleviated the reduced proliferation rate and concentrations of E_2_ or increased concentrations of P_4_ driven by MiR-125b (Figure 6C–E). Overexpression of SREBP also reversed MiR-125b-driven inhibition of proliferation rate and E_2_ concentration or promotion of P_4_ concentration (Figure 6F–H). In addition, we found MiR-125b overexpression significantly decreased the protein expression level of SCAP and simultaneously downregulated SREBP protein, and the SCAP and SREBP overexpression efficiency were shown in Figure 6I,J.

Next, luciferase reporter plasmids containing the SCAP-WT MRE motif or the mutated versions were constructed and co-transfected with MiR-125b mimics into GCs (Figure 6K). MiR-125b significantly reduced luciferase activity of the reporter containing the MRE-WT motif, while SCAP-MT luciferase reporter activity was unchanged under the MiR-125b overexpression (Figure 6L).

### 2.7. SCAP Is Involved in GCs Proliferation and Steroidogenesis via Activate SREBP

**Figure 7:**
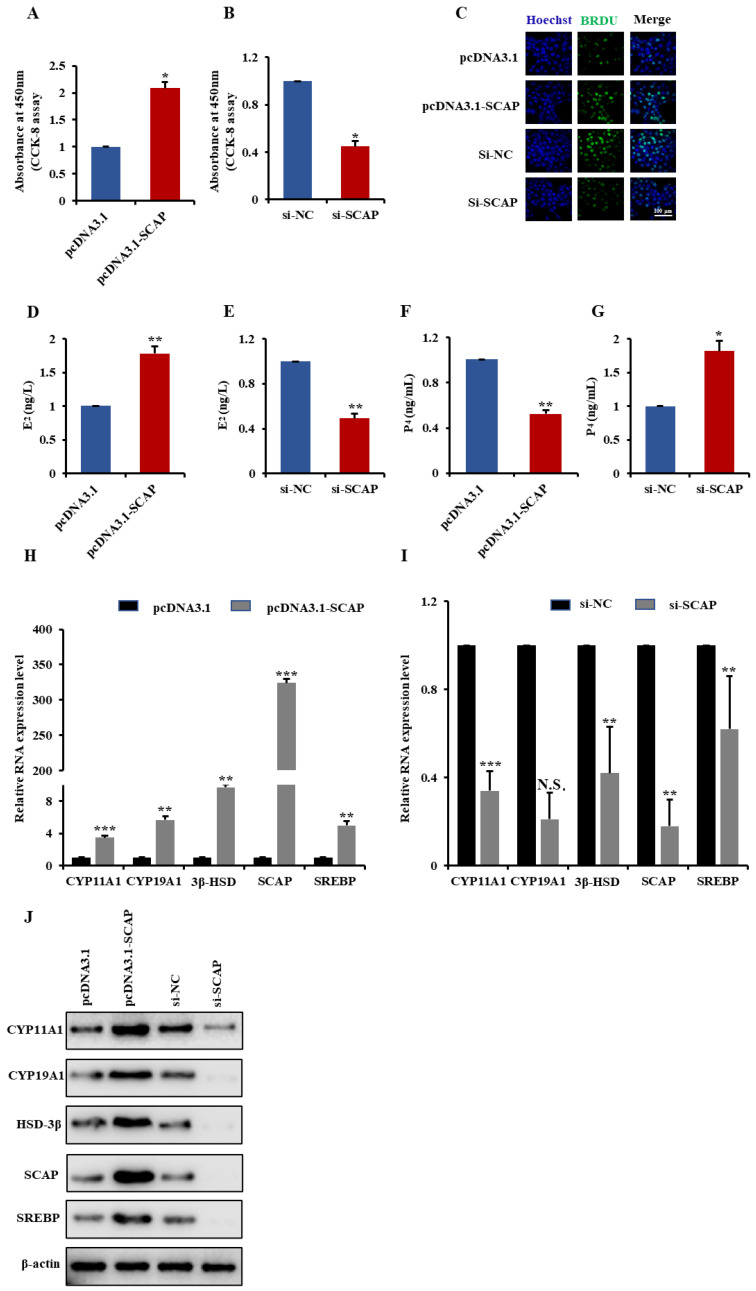
SCAP promotes GCs steroidogenesis and proliferation. (**A**,**B**) CCK-8 assay was performed to assess the effect of SCAP overexpression and knockdown on GCs proliferation. (**C**) BrdU analysis was performed to assess the effect of SCAP overexpression and knockdown on GCs proliferation; scale bars are 100 µm. (**D**,**E**) Detection of E_2_ concentrations in GCs transfected with pcDNA3.1-SCAP and pcDNA3.1 empty plasmids, i.e., si-SCAP and si-NC. (**F**,**G**) Detection of P_4_ concentrations in GCs transfected with pcDNA3.1-SCAP and pcDNA3.1 empty plasmids, i.e., si-SCAP and si-NC. (**H**,**I**) RT-qPCR was used to examine the effect of overexpression and knockdown of SCAP on the expression levels of steroidogenesis-related (CYP11A1, CYP19A1, and 3β-HSD) and sterol regulation-related (SCAP and SREBP) mRNAs in GCs. (**J**) Overexpression and knockdown of SCAP affected the expression of steroidogenesis-related proteins and sterol regulatory element proteins. Values represent means ± SEM for three individuals. * *p* < 0.05; ** *p* < 0.01; *** *p* < 0.001. N.S., not significant.

We investigated the potential functions of SCAP in GCs. Here, we transfected SCAP overexpression vector (pcDNA3.1-SCAP) or SCAP small interfering RNA (si-lncGSAR) into GCs. The CCK-8 assay and BrdU staining showed that overexpression of SCAP significantly promoted GC proliferation rate compared with that of the control (Figure 7A,C). Conversely, proliferation rate was significantly inhibited after SCAP KD (Figure 7B,C), indicating that SCAP promotes GCs proliferation.

To assess whether SCAP affects steroid hormone levels in GCs, we measured the concentrations of E2 and P4 in GCs after transfection pcDNA3.1-SCAP or vector control for 48 h by ELISA. The result revealed that overexpression of SCAP significantly increased concentrations of E_2_ (Figure 7D) but decreased concentrations of P_4_ (Figure 7F). In addition, we found that SCAP overexpression significantly upregulated expression levels of steroidogenesis-related (CYP11A1, CYP19A1, and 3β-HSD) and sterol regulation-related (SCAP and SREBP) mRNAs (Figure 7H). Western blot analysis also showed that overexpression of lncGSAR significantly improved the expression levels of steroidogenesis-related proteins and sterol regulatory element-binding proteins (Figure 7J). On the contrary, SCAP interference inhibited E_2_ secretion (Figure 7E) but promoted P_4_ secretion (Figure 7G) as well as significantly downregulated mRNA and protein expression levels of steroidogenesis-related and sterol regulation-related genes (Figure 7I,J). Together, these results suggest that SCAP is involved in GCs proliferation and steroidogenesis via activate SREBP.

### 2.8. LncRNA GSAR Promotes GCs Proliferation and Steroidogenesis via Activating the SCAP-SREBP Pathway by Acting as an MiR-125b Sponge

**Figure 8:**
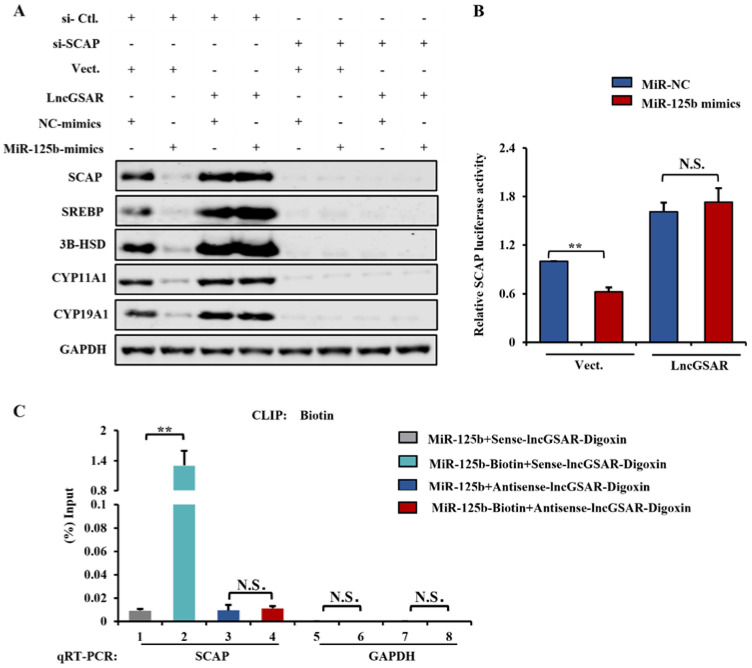
LncGSAR functions as a ceRNA to regulate SCAP-SREBP pathway by sponging MiR-125b, thus promoting GCs proliferation and steroidogenesis. (**A**) Western blot detection of steroidogenesis-related and sterol regulatory element proteins. (**B**) Dual-luciferase reporter demonstrated the binding relationship among lncGSAR, MiR-125b and SCAP. (**C**) The interactions of lncGSAR with MiR-125b and MiR-125b with SCAP were determined by RNA-RNA interaction pull-down assay. Values represent means ± SEM for three individuals. ** *p* < 0.01. N.S., not significant.

The established binding mechanism between MiR-125b/lncGSAR and MiR-125b/SCAP inspired us to further determine how lncGSAR regulates the direct targeting of MiR-125b to SCAP and whether lncGSAR regulated steroidogenesis-related proteins’ and sterol regulatory element-binding proteins’ expression is dependent on SCAP. Therefore, we firstly performed Western blot and dual-luciferase assays on GCs after co-transfection of lncGSAR, MiR-125b, and specific siRNA of SCAP. We found that overexpression of lncGSAR could restore the inhibition of steroidogenesis-related proteins’ and sterol regulatory element-binding proteins’ expression exerted by MiR-125b. Meanwhile, lncGSAR regulates these proteins’ expression, which is dependent on its regulation of SCAP (Figure 8A). Additionally, a dual-luciferase reporter analysis similarly demonstrated that lncGSAR blocks the direct binding of MiR-125b and its target SCAP (Figure 8B).

To further explore whether lncGSAR affects the binding of MiR-125b to its target SCAP, we labeled MiR-125b and lncGSAR with biotin and digoxigenin, respectively, as well as performed RNA-RNA binding assays. We found that biotin-labeled MiR-125b was associated with SCAP but not unlabeled MiR-125b. Furthermore, digoxigenin-labeled-sense-lncGSAR disrupted the binding between MiR-125b and SCAP but not for the digoxigenin-labeled antisense lncGSAR (Figure 8C). Taken together, all the results support the idea that lncGSAR acts as an MiR-125b sponge to protect the SCAP from the attack of MiR-125b, thereby promoting cell proliferation and E_2_ secretion and inhibiting P_4_ secretion.

## 3. Discussion

Ovarian follicular development is a highly coordinated and nutrition-sensitive process [23], and glucose is an important energy substrate for metabolism in the follicles of many species [24,25,26,27]. Previous studies confirm that short-term dietary supplementation of ewes during the luteal phase can increase fertility due to elevated glucose and steroid hormone concentrations in follicles [19,28,29], whereas glucose has deleterious effects on ovary structure and function at higher concentrations [5,30,31]. Our previous study also demonstrated that glucose dose had a significant effect on GCs function, with 8.4 mM representing the optimal glucose concentration for GCs to secrete steroid hormones in vitro and a higher concentration of glucose (33.6 mM) inhibiting GCs proliferation and steroidogenesis [6,7]. Therefore, there is a potential regulatory relationship between glucose metabolism and steroid hormone synthesis, and further investigation is required to validate the molecular mechanism of glucose-induced GCs differentiation and steroid hormone secretion. In the current study, we identified a candidate lncRNA (lncGSAR) involved in ovine follicular steroidogenesis and firstly reported the expression, function, and mechanism of lncRNAs in glucose-induced GCs steroidogenesis. Therefore, this highlighted the importance of lncRNAs in regulating ewe fertility, which may provide new perspectives on the molecular mechanisms by which nutrition regulates reproduction in ewes.

With the development of next-generation high-throughput sequencing, multiple lncRNAs have been identified and been shown to play different roles in regulating fecundity. However, studies on lncRNAs related to the regulation of follicle development mostly come from human reproductive diseases (such as polycystic ovary syndrome [13,32] and ovarian cancer [33]), and most of them focus on the effects of lncRNAs on GCs proliferation, apoptosis, and differentiation [34]. Our study demonstrated that lncGSAR was mainly expressed in high-glucose group GCs and controlled follicular development by regulating GCs steroidogenesis. This suggests that lncGSAR is involved in sheep nutrient (glucose) stimulated follicle development and regulates GCs hormone secretion. Studies found that lncRNAs containing MREs act as molecular sponges to effectively inhibit miRNA function [35,36]. For instance, lncRNA MLK7-AS1 regulates proliferation, metastasis, and EMT process in ovarian cancer cells by sponging miR-375 [37]. LncRNA OIP5-AS1 sponging miR-34a to promote ovarian carcinoma cell invasion and migration [38]. Similarly, using bioinformatics, dual-luciferase reporter assay, and RNA-RNA binding assay, we further confirmed that lncGSAR can adsorb MiR-125b in GCs to inhibit follicular function. These studies may provide insight into the mechanisms of lncRNA regulates fecundity in sheep.

As we all know, miRNAs directly or indirectly regulate follicular function [39] and hormone secretion [40]. MiR-125b is an important miRNA, and many studies have focused on its biological functions in cell proliferation and differentiation [41] as well as cancer cells [42]. Emerging evidence suggests that in both mouse and human GCs, androgens suppress follicular atresia by enhancing MiR-125b expression, which then targets proapoptotic proteins [43]. On this basis, our study revealed that MiR-125b is involved in the regulation of GCs hormone secretion function and further identified the sterol regulation-related gene SCAP as a novel target of MiR-125b. SCAP is a protein with a sterol-sensing domain (SSD) and seven WD domains. In the presence of cholesterol, this protein binds to sterol regulatory element-binding protein (SREBP) and mediates their transport from the endoplasmic reticulum to the Golgi apparatus. SREBP is then proteolytically cleaved and regulates sterol biosynthesis [44,45,46]. In present study, we also found that the SCAP/SREBP signaling pathway can regulate steroid hormone secretion in GCs by enhancing the expression of steroidogenesis-related (CYP11A1, CYP19A1, and 3β-HSD) genes, which is consistent with the role of lncGSAR and inverse to that MiR-125b. Meanwhile, our results showed that overexpression of MiR-125b reduced the protein levels of SCAP and SREBP. Luciferase reporter gene analysis also confirmed that SCAP is a target gene of MiR-125b. These observations suggest that MiR-125b regulates GCs steroid hormone secretion by targeting SCAP. Furthermore, lncGSAR overexpression increased proteins’ expression of SCAP and SREBP, whereas this effect was eliminated by MiR-125b mimics. The dual-luciferase reporter assay and clips test also confirmed again that lncGSAR acts as an MiR-125b sponge to protect the SCAP from the attack of MiR-125b. This result further demonstrated that binding of lncGSAR to MiR-125b regulates GCs state and function by targeting SCAP. However, there are various models for lncRNAs’ functions, and the regulatory mechanism of lncGSAR in vivo requires further study.

## 4. Materials and Methods

### 4.1. Cell Collections and Culture

All procedures used in this study were approved by the Animal Care and Use Committee of Hebei Agricultural University (Hebei, P.R. China; permit number 2022100). Fresh ewe ovaries (from thin-tailed Han sheep, ages ranged from 1 to 1.5 years) were collected at the local abattoir (Baoding, Hebei, China) and transported to the laboratory within 3 h in a buffered saline solution supplemented with streptomycin/penicillin mixture (1%) maintained at 37 °C [47]. Small, immature follicles between 1 and 3 mm in diameter were punctured with a disposable syringe, with follicular fluid collected from numbers of ovaries (ovaries number > 50) to negate any individual animal effects. The follicle suspensions were pooled, and GCs were harvested immediately after centrifuging at 1000× *g* for 10 min. GCs were counted with a hemocytometer (Axio Vert. A1, Zeiss, Oberkochen, Germany). Then, the GCs were seeded in cell culture plates (Thermo Fisher Scientific, Waltham, MA, USA) at a density of 2 × 10^5^/well and cultured in Dulbecco’s Modified Eagle Medium (DMEM/F12, Gibco, Carlsbad, CA, USA) supplemented with 10% fetal bovine serum (FBS) (Gibco, USA) and 1% streptomycin/penicillin mixture in a humidified atmosphere at 37 °C and 5% CO_2_ for 48 h with the medium changed every 24 h.

### 4.2. The Granulosa Cell (GC) Model of Glucose Treatment

When cell confluence reached 80% medium, the original medium is removed. Cells were washed with 1 × PBS, and then, all treatments were cultured in DMEM, which was prepared with no serum, glucose, pyruvate, and phenol red (Solarbio, Beijing, China) for 8 h. Subsequently, the cells were supplemented with 8.4 mM and 33.6 mM of glucose cultured for an additional 24 h. The 8.4 mM represented an optimum glucose concentration for the secretion of steroid hormones by ovine GCs in vitro [6,7], and the 33.6 mM group represents 30 times the physiological concentration of glucose in follicular fluid and was used to detect changes in steroid hormones at super-physiological concentrations [48,49,50]. This culture system was developed so that GC retains hormonally responsive aromatase activity and does not luteinize with time in culture [51,52,53,54]. The GCs were collected for subsequent RNA-seq after the 24 h treatment period.

### 4.3. RNA Sequencing (RNA-Seq) and Bioinformatics Analysis

Ovine GCs subjected to optimum groups (*n* = 3, add 8.4 mM glucose groups) and high groups (*n* = 3, add 33.6 mM glucose groups) were used for RNA-seq. The total RNA of each sample was isolated using TRIzol reagent (Invitrogen, Life Technologies, Carlsbad, CA, USA). RNA integrity was assessed using the RNA Nano 6000 Assay Kit of the Agilent Bioanalyzer 2100 System (Agilent Technologies, Santa Rosa, CA, USA). Library preparation and Illumina sequencing analysis were performed as previously described, and putative lncRNAs were screened using unknown transcripts [7].

To investigate interactions between lncRNAs and mRNAs, we constructed a complementary pair network comprising mRNA and lncRNA using Cytoscape 3.6.1(By the National Institute of General Medical Sciences, USA) (https://cytoscape.org/, accessed on 11 September 2018) [55]. Heatmaps were generated by using the R package. The raw sequencing dataset supporting the results of this study was submitted to NCBI BioProject (PRJNA825818) (https://www.ncbi.nlm.nih.gov/geo/query/acc.cgi?acc=GSE200668, accessed on 12 April 2022).

### 4.4. RNA Extraction, Complementary DNA (cDNA) Synthesis, and (Quantitative Real-Time PCR) qRT-PCR

Total RNA was extracted from cultured cells according to the manufacturer’s instructions and supplied with the TRIzol Reagent (Invitrogen, Life Technologies, Carlsbad, CA, USA); cDNA synthesis for RNA (mRNA and lncRNA) was carried out using the Prime Script RT Reagent Kit with gDNA Eraser (Perfect Real Time) (TaKaRa, Otsu, Japan). Primers were designed and synthesized by GenechemBio (Shanghai, China), and primers used for quantitative real-time PCR are listed in Table S1. Real-time quantitative PCR reactions were performed on a Bio-Rad CFX96 Real-Time Detection System using an iTaq Universal SYBR Green Supermix Kit (Bio-Rad Laboratories Inc., Hercules, CA, USA). Data analyses were performed using the 2^ΔΔCt^ method. β-actin was used as an internal control for mRNA and lncRNA, and U6 was used as an internal control for miRNA.

### 4.5. Nuclear and Cytoplasmic RNA Fractionation Assay

For nuclear and cytoplasmic RNA separation, 1 × 10^6^ cells were collected and extracted using a Paris kit (Life Technologies, Pleasanton, CA, USA), according to the manufacturer’s instructions. U6 and GAPDH were used as positive controls for the nucleus and cytoplasm, respectively.

### 4.6. Plasmid Construction, RNA Oligonucleotides, and Cell Transfection

The granulosa cells were incubated overnight (at 60–70%, confluence), and transfection or co-transfection was performed using Lipofectamine 2000 (Invitrogen, Shanghai, China) for 48 h. The SCAP overexpression construct was generated by amplifying the SCAP coding sequence, which was subsequently integrated into the HindIII/KpnI restriction sites of the pcDNA3.1 overexpression plasmid (named pcDNA3.1-SCAP). The SREBPs overexpression plasmid was generated by amplifying the SREBPs coding sequence, which was subsequently integrated into the HindIII/KpnI restriction sites of the pcDNA3.1 overexpression plasmid (named pcDNA3.1-SREBPs). The lncRNA GSAR sequence was amplified by PCR and then subcloned into the pCDNA3.1 vector, generating pCDNA3.1-lncGSAR. The empty pcDNA3.1 vector was used as control plasmid.

MiR-125b binding sites in SCAP 3′UTR or lncGSAR were amplified by PCR using a cDNA template synthesized from total RNA. Then, the PCR products were subcloned into XhoI/XbaI restriction sites in the pmirGLO dual-luciferase reporter vector to generate the pmirGLO-Luc-SCAP reporter and pmirGLO-Luc-lncGSAR reporter.

The MiR-125b mimics, mimic-negative control, siRNA target against the SCAP gene (si-SCAP), siRNA target against the lncGSAR gene (si-lncGSAR), and siRNA nonspecific control were designed and synthesized by RiboBio (Guangzhou, China).

The GCs were incubated overnight (at 60–70%, confluence). All transient transfections were performed with Lipofectamine 2000 reagent (Invitrogen, Carlsbad, CA, USA), according to manufacturer’s direction. The primers and oligonucleotide sequences used in this study are listed in Tables S1 and S2.

### 4.7. CCK-8 Assay

Primary GCs were seeded in 96-well plates and cultured in growth medium. After being transfected, cell proliferation was monitored using a TransDetect CCK (TransGen Biotech, Beijing, China), according to the manufacturer’s protocol. Absorbance was measured using a Model 680 Microplate Reader (Bio-Rad, Hercules, CA, USA) by optical density at a wavelength of 450 nm.

### 4.8. Flow Cytometric Analysis

For the flow cytometry analysis of the cell cycle, GCs were seeded in 12-well plates. When the cells grew to a density of 50% confluence, they were transfected with overexpression plasmids. After transfection for 24 h, the cells were collected and fixed overnight in 70% ethanol at 4 °C. Subsequently, the fixed cells were stained with a 50 µg/mL propidium iodide solution (Sigma Life Science, St. Louis, MO, USA) containing 10 µg/mL RNase A (Takara, Japan) and 0.2% (*v*/*v*) Triton X-100 (Sigma Life Science, St. Louis, MO, USA) and then incubated in the dark at 37 °C for 30 min. Flow cytometry analysis was performed on a BD Accuri C6 flow cytometer (BD Biosciences, San Jose, CA, USA), and data were processed using the FlowJo7.6 software (Stanford University, Stanford, CA, USA).

### 4.9. 5′-bromo-2′-deoxyuridine (BrdU) Detection

BrdU (Sigma, #B5002) incorporation assay was carried out by following the protocol. Briefly, BrdU was diluted to a final concentration of 0.03 mg/mL with fresh DMEM and then applied onto the cells grown on slices. Cells were incubated with 1.5 M HCl followed by 5-min fixation in 70% cold ethanol. Fluorescence staining with anti-BrdU antibody was then conducted. The slices images were captured by a Zeiss Axio Observer confocal microscope.

### 4.10. Quantification of E_2_ and P_4_ in Culture Medium

Granulosa cells were seeded in six-well plates and transfected with overexpression plasmid, siRNA, or miRNA mimics for 48 h, and the medium was collected for subsequent measurements. The E_2_ concentration of the culture medium was analyzed using a commercial radioimmunoassay (RIA) kit (H102, Nanjing Jiancheng Bioengineering Institute, Nanjing, China). The P_4_ concentration was also measured using RIA kit (H089, Nanjing Jiancheng Bioengineering Institute, Nanjing, China).

### 4.11. Western Blotting

Granulosa cells were seeded in six-well plates and transfected with overexpression plasmid, siRNA, or miRNA mimics for 48 h. Cells were harvested, washed with 1 × phosphate buffered saline (PBS), and lysed in RIPA lysis buffer. Equal amount of proteins were separated at sodium dodecyl sulfate-polyacrylamide gel electrophoresis (SDS-PAGE). After incubation with the indicated primary and secondary antibodies, signals were visualized by ECL. Membrane was then ready for scanning by Image studio system. Protein quantification was conducted by ImageJ software. The primary antibodies used were anti-CYP11A1 (1:1000; catalogue no. ab67355, Abcam, Cambridge, UK), anti-CYP19A1 (1:1000; catalogue no. ab18995, Abcam, Cambridge, UK), anti-SCAP (1:1000; catalogue no. ab190103, Abcam, Cambridge, UK), anti-SREBP1 (1:1000; catalogue no. ab3259, Abcam, Cambridge, UK), anti-Argonaute-2 (1:1000; catalogue no. ab186733, Abcam, Cambridge, UK), and anti-β-actin (1:10,000; catalogue no. 66009-1-Ig, Proteintech, Chicago, IL, USA). The goat anti-rabbit IgG (H + L)-HRP (1:5000; catalogue no. ab6721, Abcam, Cambridge, UK) were used as a secondary antibody.

### 4.12. Dual-Luciferase Reporter Assay

Approximately 3 × 10^4^ GCs were plated onto 24-well tissue culture plates 24 h before transfection. Cells were transfected with a mixture of Renilla luciferase and indicated luciferase reporters using Lipofectamine 2000 (Invitrogen, Carlsbad, CA, USA). Forty-eight hours after transfection, the cells were harvested and subjected to an assay by using the Dual Luciferase Reporter Assay system (Promega, Madison, WI, USA). The luciferase activity was detected by Fluorescence/MultiDetection Microplate Reader (BioTek, Winooski, VT, USA). The relative luciferase activities were normalized with the Renilla luciferase activities.

### 4.13. In Vitro RNA-RNA Interaction Pull-Down Assay

Biotin-labeled RNA was synthesized in vitro using Biotin RNA Labeling Mix (Roche, St Louis, MO, USA, 11685597910). After treatment with RNase-free DNase I, Biotin-labeled RNA was heated at 70 °C for 15 min followed by 2 min incubation on ice to recover the secondary structure of RNA. The RNA was then incubated with streptavidin agarose beads (Invitrogen, Carlsbad, CA, USA) overnight. The next day, the RNA-RNA complexes were pulled down and collected by streptavidin agarose beads. After that, the immunoprecipitated RNA was eluted, isolated, and reverse transcribed to cDNA for the subsequent qRT-PCR analysis.

### 4.14. Statistical Analysis

All experiments were performed at least three times. Data are presented as means ± standard error of the mean based on three independent experiments. All data were normally distributed, and variance was similar between the statistically compared groups. Statistical analyses were performed using SPSS version 22.0 (SPSS Inc., Chicago, IL, USA). Statistical differences were determined by one-way analysis of variance (ANOVA). Tukey’s test was used to estimate the significance of the results. A *p*-value < 0.05 were considered statistically significant.

## 5. Conclusions

In conclusion, we identified a novel lncGSAR that acts as a sponge for MiR-125b to activate the SCAP/SREBP pathway, resulting in promoting granulose cell proliferation and steroidogenesis. Therefore, this study identified a candidate lncRNA (lncGSAR) involved in ovine fecundity, providing insights into the regulatory mechanisms by which glucose regulates follicular development and a basis for new strategies regulating animal reproduction by nutrients.

## Data Availability

The datasets used during the current study are available from the corresponding author on reasonable request.

## Supplementary Materials

The following supporting information can be downloaded at: https://www.mdpi.com/article/10.3390/ijms232012132/s1, Table S1: Information of Primers; Table S2: Oligonucleotide sequences in this study; Table S3: The information of differentially expressed 461 lncRNA transcripts (281 upregulated and 180 downregulated) in the optimum and high group; Table S4: The information of differentially expressed 94 miRNA transcripts in the optimum and high group; Table S5: The information of differentially expressed 796 mRNA transcripts in the optimum and high group; Table S6: The information of ceRNA (lncGSAR–miRNA–mRNA) Network; Figure S1: Gene ontology term enrichment analysis of differentially expressed lncRNA target genes in different glucose treatment groups; Figure S2: Kyoto Encyclopedia of Genes and Genomes enrichment analysis of differentially expressed lncRNA target genes in different glucose treatment groups; Figure S3: RNA sequence of lncGSAR.
