# Supplementary material for: LncGSAR Controls Ovarian Granulosa Cell Steroidogenesis via Sponging MiR-125b to Activate SCAP/SREBP Pathway
Source: Int J Mol Sci. 2022 Oct 12;23(20):12132. doi: 10.3390/ijms232012132 (PMC9603659; doi:10.3390/ijms232012132)
Supplement: Supplementary file 1 [file ijms-23-12132-s001.zip › Figure S1, S2 and S3.pdf]

LncGSAR controls ovarian granulosa cell steroidogenesis via sponging MiR-125b to activate SCAP/SREBP pathway

Yong Wang [1], Yunxia Guo [2], Chunhui Duan [1], Junjie Li [1], Shoukun Ji [1], Huihui Yan [1], Yueqin Liu 1, Yingjie Zhang [1, *]

[1] Laboratory of Small Ruminant Genetics, Breeding and Reproduction, College of Animal Science and Technology, Hebei Agricultural University, Baoding 071000, PR China

[2] College of Life Science, Hebei Agricultural University, Baoding 071000, PR China

* Correspondence author at: Hebei Agricultural University, Baoding 071000, Hebei, PR China. Tel.: +86-31-2752-8366; Fax.: +86-31-2752-8886; E-mail address: zhangyingjie66@126.com (Y. Zhang).

Figure S1. Gene Ontology (GO) term enrichment analysis of differentially expressed lncRNA target genes in different glucose treatment groups.

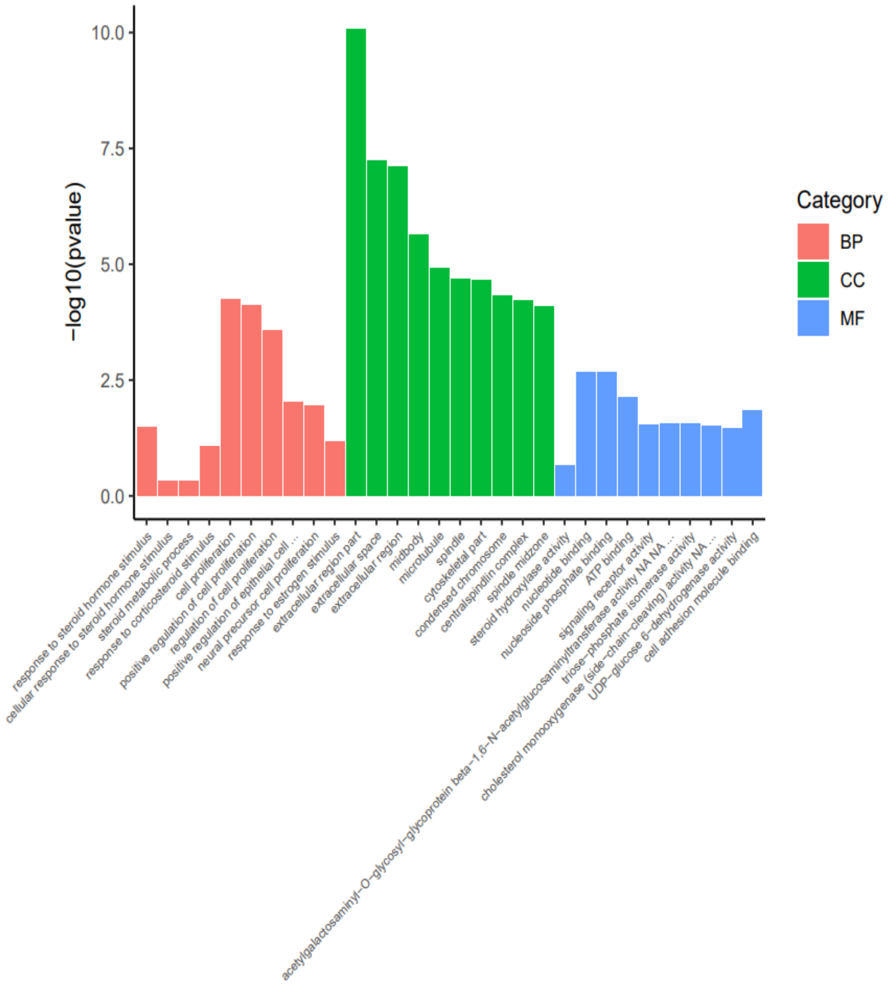

Figure S2. Kyoto Encyclopedia of Genes and Genomes (KEGG) enrichment analysis of differentially expressed lncRNA target genes in different glucose treatment groups.

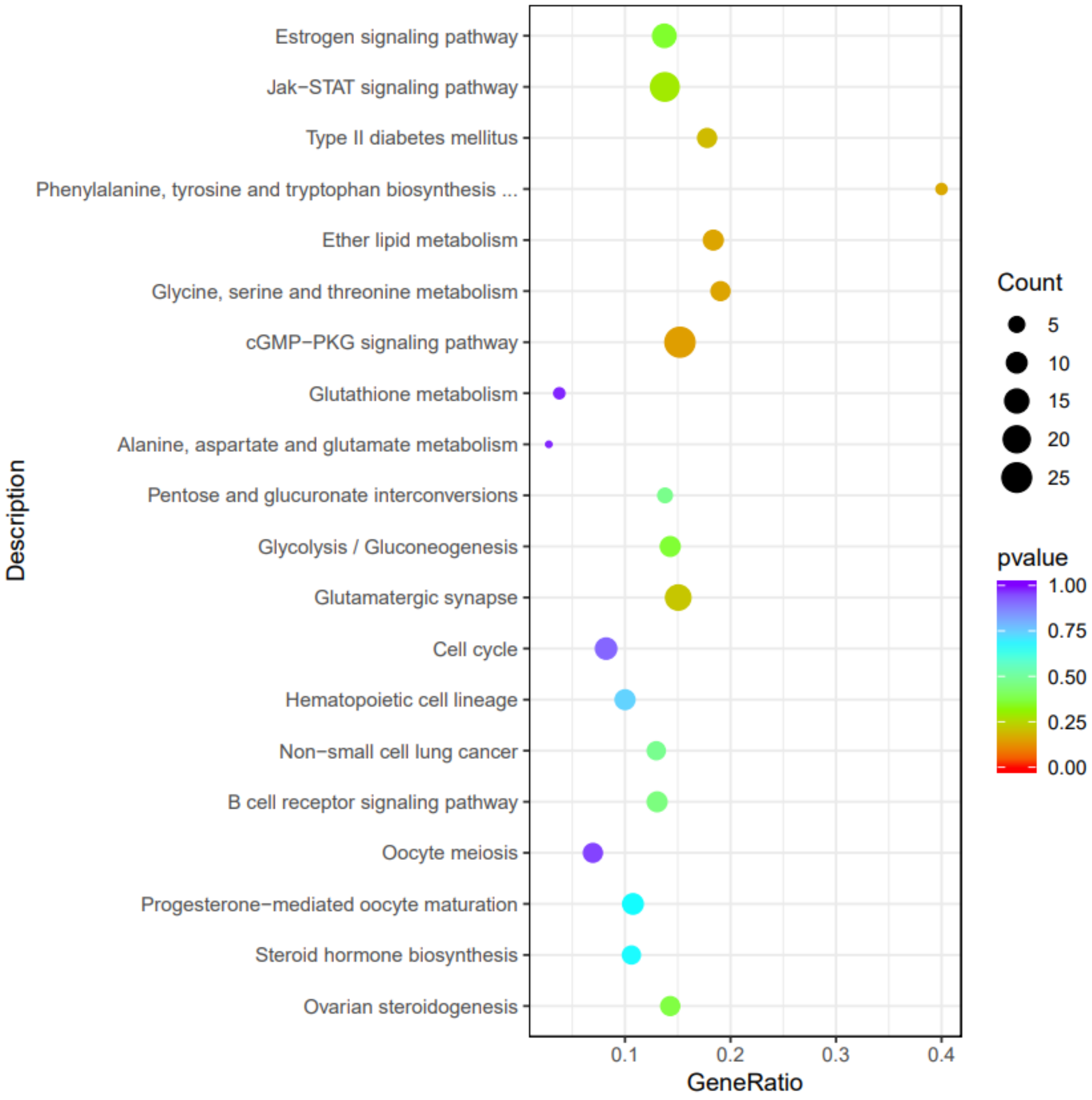

Figure S3. RNA sequence of lncGSAR.

GGTACGGAGCGGCCCGGTGCCGGGGCGGGGACGACCTGGGACGGCGGCCGCGGGCTCTGCCCAGAGTGCGG
GTGGCTGGCCGGCGGGGCGCGGGTCCGGGGGCGCCGGGCTGCGCTGGGGAAGGGGCACCGAGGGTTTGGGA
ATCGTCCTGGCATGGAGACGCAGCCCCAGAGTGCGGCCACGGGATGAGGGTGTATCATCGGAGAGTGTGTTTTG
GTGGGTCCACGCGCGTCTGCGTGCCTGAAAATTGCGTAACTTTGAGGACTTTCCCGAGACCTCTGTAAGTATAAT
GGTCAGCTTTGTTTGCCCCGGATTTTAAAAGCGGAATCGAGAGCTGACTCCTGGGTGACCTCAGATGATTGGGAC
CCGGAGGGTCAGCACTTAGTGCTGGGTCCGGGGCTGGAAGTTGTGGGGTGTGTGTTTGTGTGGTTTAGGGAAGA
AACTCGGGGAAAGTTCTCATGAAAAGACACCTTCCGTTCCCTGCCCCCCCCAACCCCCACAGTTTCTATGCGAAA
AGTTCTCGGTGAAATATCTTTCCCAGCTAAGGCGTTTTGGATTAATAGATAACTCATCAGCCGCATTCTTCTCTGAT
GAGTCAAAGACCCCGAACCTGGCTGATTAGGTTTTGTAAATTTAGGGGCGTTGCGGTTGTGGGGCGCAGCATGC
TGGAGTTTCAGACTTTGCAACTCGCGAAGTGTTGATCCAGACTGTTTTAATAGGACTTTGACGTCTGAGCGTAAAT
TTTATGTGGTGCGTCCCTAACAACGGTAAAAATGTTGCGGCTGCAGAGCGTTCCTTTGGTAGAAACAAAACTCAG
TACATGTCGGGGAACTCCTGAACAATCCTTTCAGAATTTTGTAGTTGGGATATTTGCGTTATCTTGTTAAATGGGG
CTCTGTACTTGTAGGTGCATTATCTAGAACCCAGTAAACACTGTTTTCCGCCTACCTTTTGAAAAGCCCAGGAAAT
TGACTTCTGAAACATTCCAAAAGGAACTTAAGAGTATGTGTAGATGCAAGAAAGTATGCAGTCTAGATAAAAGCA
TTCTTACAGAAAGGTGAACTGATTTCTAAGCGGTTAGGGTGTTGAGATGATTATGGAAATACTATGTTGTGTGTAA
ACTAAAATTGTGATTGTGAAGCCCAAGGTTGCTGAGGACTTAATACACTTAAGCCCTCAGCACTTGTAAAATGGG
AACTATTTAAATGGAGAAGACAGGAGAGCTCCAAGACCAAGCCAAATATTATGCAAAATAACACAAGATTTTACT
CTTAACACCGTTGTTCGTGAGTGCAACCCAGGTGAAATGCTGTTGAGCCTGACGTTGCCGTCTCACCTGAGCAAA
TCGCCTTTATTATTATAAACTGTTCTGAAAAGCATTATTTTTACATGCTGGTTTTTTAAATTGCAAGTGATGGAATAG
TACTGACATTTCTTCTCTTTCAGAAAGATGGAGACTTGAATTGCAAGTTCTGCTTTTTTTCTTGAACCTCACTGGCT
ACTTTCCTATTTTATTTTCCCTACCACTGAAATAAAATTTAAAAGGTAGTTTCTTTTTTTTTAAATGCTGGAGTATTTT
ATTTTTTATAATTAATAAAAAAGGTAGCTTTATTTAAGGAGTTGCAATGGCTTCAGAAACCTTTCTGTTTCATTAGA
TTGGCGATCTGTTGACTTCTAAGGGTAAGACTGTATCATACATATGAAACCATCTTTCTCTCTTCACCAGAAAGAG
AAACTTGTAAAAGTTCATTAGGGTAAACCAAAGACTATTTTCTTTTTTTTTTTTTTTTTTTGGCACACCATAAACTATT
TGACAGAATTAAGCATAGAAAACTCAGGTTTTTCTGTTTGACCCAGCTCAGCTTAACTGCCTGCAGCACTGGCCT
GTTTGCCCAAACCCTGGGTGTCTTTATATGTGCGAGGTTGGGTACCGGTAAAATCACAGGTGATCCCAATACAAT
GGCTGTCTCTTAACCTGTCCTATTGCCTTGTTAAGTCTTCCTTTATCAGGTGGAAATGGCCCGTGGTTCTAGAAAGT
ATCTGTCTTTATAGCTAGATACTTGTTATTTTAAAGATAAGGGGACAGATTAAAAAAATAGAACCCAAGCTGTACA
AAAGATGGAATAGACAAAACAGATACACCGTGAAGGGAGAGGTTTTATGATGTTAAAAATTTGGTAAAAGTTGG
CCTGAATGATCTCGATAATTTTGCCCTAATTCCCTGTTTGGGGTTTATGTTTTGTTTGTGAAAAACTCCTCCTTTTTT
CCCCAACCCTGAAAGCCACAAGTAAAACACATGTGTTTGAGTCCCATTAATGTGCTCAGCAATATACAAAACCAC
CCAAATGGTCCTTTCCCTTGCCCACCCAGGGATCAGCAGTTTGTCCTCAAACACATTGGAAAGCCGCGGTGGAG
GATTCCAGTAAACAGAACACACATATAAATGTATATACACAAAATGATGAGTGTTTTGTAGCTTCTGGTAGCCATT
GAGCTGATGAGTCAGTTCAGTCATCTTTGAGGGAAAATAGTTCATTTCCTAGATCATCAGGGTTATTTAGACTTTG
ACAAAATCCAGGTTTTATTCTATCCCCTCCCCCTCATTTAAGTGTGTTCCTTTTATCTTCTGGTACATATCAGCGGC
CCACCTCCTGGTCTGAGAGCTTCTGAAGCTCACTCTGAAACAATGATGCTGAAACACTTTCTTTAGTTCCATTGCT
ATAGTAGGAACAGTCTTGAGACCTAGGAGCATTTCTGCAGCTTTTTAAGTAGGGTTGGAAGTTTTTGAGACTGCTT
TGGGTAATATTTAAAACTTTGAGGAAAAATAATTTTAAGACTTGGCTAGCCAATAGTGTGAGAGAGAGGATACAT
CTGGCTTTCTGGCTTCTAGTGTGCTGTACATGCGTATTCCTGTGCAACTTGTCCGGGTAAAGAAGAACCGGTTGTC
AAAATCTTAATGGCGTCTGAGATTTGCAAGGCTTCTGTATACATTTGTGTAGGGGCAGACAGGACTGGGTGACTG
AATAACAGGGCAGTAGAGCTTTTCTTACATAGCTTTACAGTTAATAGCTTTACAGTTAATAAAAAATTCCTTGAATT
TACCTCTACTTTTGGACTAGACATTCTTCTCTTCAAATACTTCACACTTGACAAATTTCACTACTGTTAATATGTAAG
AGACATAACTTTTTGGCTTGAAGTTCTGTATATTGCAACAGTCTAGTAGTAGATATCGTTTTTGATCCCTGAATTTT

CAAAATGGAAATCAGATAAATAACTTGGTCTTATTTTTCCTTTTATGGTATGCCGTTGGACACTTTTCAGGCTGATT
ACAAGTCATTTCTAAAAGGTGTGTTATTTACGTCGAAGCACTGGTGTTAGTTTTCTCCTTAAATTGCTGTGAACGTT
CTTTAAAACAAAACCTGGCCTTGTTGGAATATTGATCAAAAAGATGTTTGGCAGATGGTGGTCAGAAATCACAAA
GTCAGGTGTTCAGAAGTTTAATACAGATCTCTACCTCGAATTCTACCTTTTAAAGTGCTTTGTAATTTTTCCCAAAT
AACTGGGGTCATGAAGAAAATTGTTTCTGATATAATTGGCACAGAGAAATGTGTGGTGGTTTGATGCGGCAACCT
CAGGTTTTCATTGAGCATTAAGATTCATCAGTATTTTGACCTGGGGAAAAATACAGAATGGTTTTGTACAGTTTAA
CCAAGTTTGGAGTCTGTTACTCCAAATCAAATCAAATACAGTAAATTTATATTTCAGTCATAAATAGGATTTGGTG
ACAGCGTTTTGGATGAAAATGTGATGATTTCATAGTAAAATGATGCTGCAAGACCACTGCCAATATGGCATGCCC
TTAGTTTTCCAGTAGTGGTTTTAAAATTTCTGTGATAAACCCTGACACTCACATGTAGTCATCTGTTTCTAAGGGCA
TTTGTGTGTTTAATGCTCTCATTGGAGATGCTTTCATGTAAATTTTGCAGGTTCATCTTAATTTTCCACTTTGAGGAT
AGAACTGTGACTGGTGATGGGGAATAAAATGGGAATTCTTGGGCGCTGCGATGAGGGCATGAATTATTGCGGCT
TTCGGAAGGACTTTGGCGGTCTGTGTCAAATTTTCAAGCCCTTTTATTCCGTGCTTTTATTTTTAGAAATTTACCCTA
AGGAAATAAGCATGCACAAAGATTTATTTATAGGATATAGACATAAATAGTCTGTGAATTTTCACTAACAAGGAA
ATTGCTAAGTAAATGGTTGTGTAATTCCTCAAAGAGAATACTATGTAGCCATTAAAAGCCACATTTTAAAAGAATG
TACCTATGTGTATTTACTTTTTTGGGGGAGGTGGCTTAAAACATTTATTCTCTCAGTTTTAGAGGCTAGAAATCTGG
AATCAGTGTTTACCTCTCTTTGAATGCTGTAGTAGAGAATCCGACTTTCTCTTCCAGCCTCTGGATTTTAATTAATT
CCCATGACATTACATGAAAAAAGTAAAAATAAAAGTTTGCTATTATGTGATGCCAATTCTTAAAAATAATTGGATA
TGAATACAAACAGTGTGGTGGATTACAGGTCTTTGTATTTACCTTCTGTTGCTTTTCTGGATTCTCTAAATTTTCTGT
AATGAACATATAGGCACAGAAGCAGTTCTAGGTCTTTGCTTAGAAGAAATCATTAAATACCCTTGCAGTATCTATA
GGTGTCACAAAACCATTGTTGGCCACGTTTCATTTTGCTCGCAAGCCTTGCCCCAGGAATCAGGCCTCCATATCTC
AAAGCATGTTTACCTCGCTCATTTCCTTGTCCTGTCTGTTCCTAGTGATCTTAACCTGCTGTCCTTGTTCTGATGTTC
TTTCTCAATCCATCCACACGTACACTGCTGGATGCTCTGGTGCCCAACTCCGTCAGCACTTGGTGAAGTTTAAATA
TGACCTTCAAGATTGCTTTTGGTGCTCTGTAATATTCTGCACACCATTTATTTCAACCAGTTTTCCCCTCTTGGTCAA
GCTTGAGCTGCTTTCTAGGCAAAAGCCAAGGTTCAGCCTGCATTTTGCAAGATCCCATCACCTGAATGTCTTATCC
CAAAGAGATGACTTTCCTCAGAATTCCAGAAGTTATATTCTTCTAGTTAACTCGTGGATCATTATCTTTAAAAAAAT
AAACAAAAAAAAAAACCCTGCCATCTTACCAGAGTATTTCTAATCCCCAGCACCTCCCCAGGTAAGTACCACCCAT
ACACACTTAAACCTGTGTGCACATATAATTCTTGATAAACAACTTATGAACATAGGAAATATCCTTACTGGTAGGA
GTGGTAGTGATGACCTTGAATAGGTAAACTCTCAAAAAGCAGCCACTTAATAACTTCATGTGCAGTTTAGAGTCT
GTGAAGGATGGACCCCCCACCACCCTCCAAAGTGACCTCCCCAACTTGTCACCTGCAGCCTCCCTTTGTGCCCAG
AGGCCAGTGTGCCTGGTCCATCTAACAATGTGGGCCCGCTCATCTCCAGGTGCAGCTGGGTCTTCACTGTTAGAA
TGTGGTGTCTGCTGTGGGATCCGTTCTGAGCTTTTGGACCAGGGCTTCTGGGAGCTCCAGTTCGATGTGAGAGAA
GCATTCCCAAATGAGCCTGCTCTGTGTGACCTGTGTGACTGCCACTTTGAAATGTTTGTGTTCTTTTTCATTTGGCA
CCTTAGTGTCTGCCCGGGGGTAGGGGAAAGGGAATGCAAATGGTCCTCTTGAACAGTGCTTGCTTTGGTGAAAG
GAAAAATTTAGGAAGGAATGAAGAGAATGAGCCTTAGTAATAGCTGGGGCAGAAGTGGCAGCCTGGCTCTGTG
GGGCCTGAGGCCAGCTGGGACCAGGCCTTTAAAGTTGATTGGAGGGTGTCATCGGGAATGACCTTGTCTGCCCT
GTGGGGATAACTTGCACTGACTCACGTGGGTCCCTTTAAGTGTGGGGAGGATATTGCAAACCACTTCAGTGTGTT
GTGTGTGTTGGGGGCAAGTGGGGAGTGCTTGACTGCAGAGCTGGTCAGAGCAGAGTTGCCTAGTTTGTGGCCTG
GTCATCTTTGCTGGGCGTCCCACGTGGCTCAGTGGTAAAGAATCTGCCTGCCAATGCAGGAGACTCTGGTTCGAT
CCCTGGATCACGAAGATTCCCTGGAGAAGGAAATGGCAACTCCCTCCAGTATTCTTGTCTGGGAAGTCCCATGA
GCCTGGCAGGCTACAGTCCATGGGGACGCAGAGTCAGACACGACTCAGCGACTGAGCACGCATACACATCTTT
GCTGAGTGCAGAAAAATGAAAATTCTGAACTACTTGATCCTGGGAAATTTTCAGAAATCTTTTTAGCCATTCACCC
AGGTAAGAATTATTAACTTTTGAGTATTTACTTTGTGCCGAGTACTGGCCAAGCACTTTTATATGTATCTTGTGACT
CATCCCCACAATGAGCTAATGTAGCGATCAGGGCAGTGATGAAACTCTTTCTGTGGGGAGGTGACTGAGATCCT
GAGTGCCTTGGGACCTTGAAGGTTCTGAGGAAGCCAAGACCTGGGACAAAGTTCCGACTCTTAAGTTGCTCCCA

GGTTTAGTGGAGAGGGCCTTGTGTTCAGTTAACTGAGACAGTAGTTTGAGCTGGAAACCAGTCAGCACCTTAAGG
AGGGAGAGGGTCATGGAGCTATATCAAAAGAAACTTTTTAAAAAGAGAGGTGATACTGGGATTTTTTTTTTCCCC
AGCTGAGGGAAGGATTGAGGCATTAGGGGAAAGATGGTTCAGTCAAAGGGATCGGGCCTTCTGTGAAGATACA
GAGGCTTAAGAGGTAAAACAAGCAGGGGAGAAAGGGAAGTATGGGTGATTGGGGTGGGGGAGGGATGGAGGA
GTCCCTTCCATGCCAGGGACCAGCACGCTTCCTGTGATGGGAGTGGACTTCTGTGCAAGGGGGACGTGGCATCC
TGGGAGGTTCCATTGCGGAAGTGGATGCATTAGGTTTCAAGGCTAACGCTGCAGGAATCCTGCTCTTAAAATAAG
ACAGTAGCTGCTGGTGGTGACTGAGCCTGGCAGGGGCAGGAGAGGTGTTCACGGGCTGCAGTAACTGTCGGAT
GCTCTAGAGAGGAGACTGGGAGCTCCATCCGTGGAGCCTCTTGGGTTTCTCTTTCGGATGATTAGCTGCGTGGTG
GTTGTATTTGTAGAAGCCAGAAAGTTTACCTTGAAAGGGGAAGAAAAGGCAACCGGGAGGTGAAGAGGTTGGAT
TTGAGATGCAGAGGTCTGATCTTGCAAAGAAATTTTCAGAACAATTCAAGATTATGTGGGACTTCCCTAGTGGCT
CAGGGGTAATGAATCTGCCTGCAGTGTAGGATGTGGGTTCAGTCCCTGGGTCAGGGAGATCCCCTGGAGGAGG
GCATGGCAACCCACTCCAGTATTCTTGCCTGGAGAATCCCATGGACAGAGAAGCCTGGCGGGCTACAGTCCATG
CATGGAGTTTCAAGAGAGTTAGACATAACTTAGGGACTCAACAATAACAAAAAGATTATGTAGCTGGTCATCGAA
AAGGTAAATATGAACTGAGTGCCCAGAAAATTGGAGAGGGTGTGGGACCCCAGTTCCTAGGAAACTTGCAAGG
GTAGGCAGCAAAAGATGGACAATTGACTTAAAATTGGAAACTGTTTTCTTTTAAGGCGTGGGGCGGGGTGAGGG
GGAGAGCTGGACATTCTCAGAAAAAGTTAAAGCAAAATTTTAAATGCACTCTCAATTTAGGAACACAAAAATGTC
TTGCATATAATCTGCCTAGTTATACTAGTGTTCAGTGTACTTAACCATTTGAAAGGCTGATTCGGAGGAGCCTAAG
AAATCTAGTTATATATAGTGTATTTCATATCCTTTCATTTTTTTCTTTCATCAACAGTCTTTGAACATTGTTTTCCTCT
AAAATTTGCAGTTCATGCTTAGGCTTTTACTTAAACTTTTCTTTTCCCAAACCTTTACCTAGAAGGGACATAAAATT
TGCAAATAGGTTTGGAAGGTAAAAGATGGAAGGCGTGGAGTGGCTTGAGTATTTTTATAAGAAAATCCCAAATTG
CTTTGTTCTTTTTCATTTCATAGGGGTTGGGGGAGTTAGAATGAGTCTTTGGCTAGAGCCCAAAGTCGTCTCTTCTG
TGGGTTATATTTTCAATTTATTCATTTCGTTTGCTGCTTTTTCTGCTCTCAGCTAAGTGTCAGCTTGTGAATAAAGAA
ATGCGGTCCAGGAATTTAAGAACAAACAATTGTGTTTCTCGTAGGTTTTAACCCTACTGCAACCATTCTGATGCAC
ATTATGTAGAATACGGGAGTTAAAAAAAGGAAATGGCAGTAATAAATGCCAATCACGCCAAGGAAGATGGTTCC
AAATTCCAGGGTGTAATACAGTATTGTTACACAAATTTAAAACGCTGAGGGGTTGCTAACACAGCATAGCTCGGT
TCTGTCACCCCCCTCAGACTTTGCAAAGATACAAAGAAATCTAGGCATCTCAAAATCGTAGTGTATCTCACGAGC
CCACGAAAAGAAGTTAGAGACTAATTTCTACTTCCAGTTAATCTTTACTTCTCATGAGTTTTTTCCCTTAAACTTTCT
TTGCTTTTCCTAAATTCTTATGTCACTAGGTAGATATGCTGGCAGGTGGCCAGCTTTATTCTAAGAAATTTTTTTTCC
TCAAATCTCCAGGGATCCTGTTTTGTGAACATTTATTTTTCTGATGCCCACCTGTAGGATGGATAGTCTTGGCCTCA
TGAGAGAAGGGATGGCTCAGAAAGTGCTATCTTATTACAGAAGTATCAGAATTTCCTTTGTGCTCAGAGGGGAAG
TGCTACAAAAGTGTAATTACTTTACCCAGCATGTGTGATTTGCAGGCCTGAAAATGTGCGAGTTTCCAAAAGCAT
CTGTTTTTAAATACAGTATCCCTCTAAGCCGGCAGGATGTGTTTTGGGCCAGGGAGGGGACACACACACAGCTCT
CTCCACTGTGACATACACTCACACTCCCTGTCCTGTCCTGTGCCTTCCGTGGGATGCTGGGTGACGAGAGGCTGA
AAGTGTCGGTAATTGCTTCTCTGGGCCTTGCTTATCCTGAGAAGAAACACAAGCTGTCCTGGAGTCCAGTCAGCC
TCCCCTCTGCCAGATCACTGTGTCCTGAGTCTGCCTTCTCACCTCTCTACCCTTCTAAGCCTGTTTCTTTCTTTTCTA
GGAACTCCAAGCATTTTCCCAGCAGTGAGCAAAACAATAAAAAAAGGTCAGAGTCAGTGCTGAGACCATTTTTGT
TGCAAACATATTTTTTTTTAGTCTTCACAGGTATTAAAAAAGTTGGAGGGATAATCGAAGATCAGGTGCATGGCTC
CAGTATAATCAAACTTATAAAAGCATTTATGGATTCTTGGCAGATTGATTCACTAAGCATCTGTGGACTAATGTCG
TCTCTTTTAAGCACTGTGCTTTTCGATATTTTTTCTTGGAGTATAGTTGTTTAATGATGATGTCTTACTTTCAGGTGAT
TCAGTTATCCATATACATGCATCTACTCTTTCAAATTGTTTTCCCATTTAGGTTATTACAGAGTATTGACCAGGGTC
CCCTGTGGTGTACAGTAGGTCCTTGCTGGTTATATATTTTAAATACAGCGGTGTGTATACTAAGTGTGTACTTTAGC
ACAGCGTGCACTTTGACACAATGAGGTGTTCTCATGTTTGGGGATGAGGAAACTGAGGCTCGGAGAGATTAAGT
AATTAGAGGTTTTATTTGCAAGTGGTGGTAGGAGCTGAAAATCTCTATAGTGGTATGTGAGGTTCATGTAAATCTG
GGCCTGCTGGTCTCCTTGACCTCATCCACCAGGAGTCACAGCTTCATTATGCTTCAGCTGCTGGGCCTTACTTAAG

TCTCTCCAACATGCCAAACCTGTGCTGCCTCAGGGCCTTTGTGTGTGCTCTCCTTTCTGCCTGGGAAACCATACAC
CCAGGTATGAGGCAGCCTTGTCCTTCTCATTCATTCAGATCTCAGTTCGCATGTTACCCAGAGGGACCTTTGGGTA
ACAAGTTCAAATGAGCCCCAAGTTCCCAATCACTGCCAGAGTCCTCTTATTTGCCCCCCAGGACTTCTTCCTCACT
GATATTACCTTGTGTACCTGTCTTGTACTGCTGGAATGTGACTCTGGTCTTATTTAAGGCAGTAATTCCAGTGCCTA
GAACAATGAGTGGAACTGCCCAATACATTTGTTTGTTTGTTAAATGACTGGAATACAAACCAACTTGTCAAATTCT
GAGGCCTGCGATACACTCCATCGTCCTAATCCTTTTCCAGCAGTTTTTCAGTTCAGTCACTCATTCGTGTCCAATTC
TTTGTGACCCCATGGACTGCAGCACGCCAAGCTTCCCTGTCTATCACCAACTCCCGGAGTTTGCTCAAACTCATGT
CCGTTGAGTCGATGATGCCATCCAACCAGCTCATCCTCTCTCGTCCCCTTCTCCTCCTGCCTTCAGTCTTTCCTAGA
TTTTCAGCATTTCCCAGCAGTTTTTAGATTAAATCTTAACGCTGCCTTCAAGATGATCACTTCTCGATTCCCATTTAA
TTACATCAGCAAGGACAATTAGCACGTGCTTTTAAGCCAGAAATTTTGGATGGCAAGTGATGAGTCCCATGGAGA
TGAAGAGGGTGCGAGAGAAAAGCTGATTTTCACATGTAAAGGACTACTTCCCTTTGTCCAGTAAAAATGGAAATC
GAATACATATTTCCACAGAAGCAGGAGTGAAGTGTGGGCCGCCCAGAGAGTGATAAAGTTGAGTAGAGCAGGG
TGACTTGGATTCCTGCTTTGTAGATGTGCTGTTGGTTTGCAAAGAGTTAATGGTAACAGCCCAGGTGGAATTGATT
TGGAGATAGACACACAGGCCTTTGATTCAGTCTAGCCTCCAGCTCAGAGAGCCTTCTCTGCTTTGCAAAGTGAGA
AGGCAAGCAATGCAGGGTGCTGTGGGCTTTAGCAGGCCCTGTCCAGCCTGTGAGCACCTGTGTGGAAGCCATGA
GGCCGCCGCTAATCTGAGCCCTATGAACCCTTAGGGAGGCACCTCACAGCCACTGGTCAGCCTCACAGACTCAG
GAGTTGCTTTTATTCCCTGTAAAACACAACAGATGACAGGTTAGGATGAAAGAAATCCAAGTCATCCTTACTTTGG
TCATTGGATTGTCTGTTAATCGAGCCCACGAAGGCACTCATGAAAACGCAGGCCATTGGCATATTCTTGTTTATGG
CTTTCAGGTCCAATACTTGAGCCTGAACAGACTTTTTATTTTCCTAGTTTTCTTTTTGGCTACCTGTGTGGCAGGTG
GGATCTTAGTTCCTTAACCACTGGGCCACAGCGGAATTCCAAGAGCAAACTTTTTAAACTTTGCCGTCATCCAGT
GTCTGACAGTCTTGGAACCAGTGGTATATAATCTTTATAATAGTCGATTCTTGACCAAATAAATCGTCAGTACTCT
CTAAGATCCCCTGGAGAAGGGAATGACAACCCACTCCAGTATTTTTGCCTGGAGAATCCCATGGACAGAGGAAC
CTGGAGTTGGACACTGAGTGACTAACATACACGTTGAAAAAAATTGTTATACCTTTTTTCCCTTTTGTAAAGGACC
TAGGACAAGTCACTTAAGTATCTTGTTTCCCTAACTAAAATATAGAGGTGGAGAGACAACGCTTGCTATGCATTCA
AAGAGCTGTAAGTTTTTCTATTTTATCCCACTTTTCATCTCCTCTGTGTGATGCCAAGTGCTGTGGTGGCCCGCCGG
CCCAGATACATCCGGGGTGGAGGGGGAGAGACTGTTTGGGAGGAAAGGTTTTAAGCCTGAGATTGTTTTGTTTTT
GTTTTTCTCTAAGCAAATCAGTGCCTCTTCTGGAGAACTGGGTGGGAGTTTCTGGGTGTGAGCTAACGGCAGGAA
ATGACCCTCTCTAAACAAAAGGACTCTTTAAGGCCCATCCTGAGTCTTTGTGTTTCTCGAGGAGGGGTTATGCAG
AAAACCAGAGCTAGGAAATTAATCTCTGCTTTTAGCCTTTGTAAGCGGGGGAGGGGAGAAAGAAGAAGATTCCT
TCCCTGCTCGCCCTCCTAAACGTCCAAAGGGCCCGCCAGTACCCACTGAGTGCCAGAGAGGTTTAAGAGCAAGG
CAGCTCTTTGGGCCAGTGTGCCTTCATGCCCACAACACGCAAAGAAAAACCAGGTTGGGCAGAATTACACTCAC
TGTGACCTTCAGCTTTAACCTTGGCTTTCAGCTGCCGCTTGAAAAAATCTATCACCAGCAAAATGCAGAATGGGG
AAAGGAGAAGAAAGGAATATTTTGTATGATCTATTAGTTGGGGTGCTGAAGTTTGCTAAATAACGTGAACGTCGC
TCAGTCGTGTCCGACTCTTTGCGAGTCTGTGGATAGTCCATGGAATTCTCCAGGCTAGCATTCTGGAGTGGGTAG
CCTTTCCCTTCTCCAGGGGATCTTCCCAACCCAGGGATCGAACCCAGGTCTCCAGCATTGCAGGCAGATTCTTTA
GCAGCTGAGCCACCCCCATAAATAAATCAAATATTTGAATACTACATTAACCACAGTCTTCATTTTTCTAAATCTTG
ACAGAGAAGAGCCGGTGACTTCTGCCAGTTGATGAACACAAGGCTAAGCTTTTCTGTGTTAGATGGCAAGCCCCT
ATGAAGAAGTGTTGGGCCTTGCCCCTGCTTTGGCATGCTGCTGCTCTTTAAAGTGACCGTTCATTGGATTCATGTA
TCTGTAGGGCCGAGTCCCTCTGCTGTTCACTGAAACTACCGCAACATTGTTAATTGGCTATACATCAATACAAATT
GATCTTAATGACCATTATATCAATATTTACACTGCAGGATTCTGAATTTATATTGCTAGGAAAGTGTCGATAGTTCT
GTCTTATCAAAAATTATTTTGCTTTTACAAATTCTTGCTGACTTGCAGCAATGAACTTCTTCCAGGCACACCACTTTT
ATTTTTTTTTTTAAAGGAATATAAATCTTCCACTAATAATTAGAAACACATATTTTGTTGAAAATGGTTTTGTGGTTT
TACTTCATTTTCAGAGTGAAAACGACAAATATCTTCTCCAAGAAACTTCCTCAGCCTACTATTTTAGGCTTTATATT
GTAACTGTATTTTTAGAAAGGCCTGCCGATGTGTTTGGTGGGTACAATGTGGTGGGGTGGCGGCGGGGGGGTGC

GGAGGAGGGTAGAGAGGTGGATTCTGTCACAGCACCAGACACTTAACAGAATCGCAGAGATAAGATTAAAGCC
AGTGCTTGGATCCCAGAATGAAAGGATTTTGTCTGGATGTTGCATGTGTTGAAAGACTCTGTATCTCAGATAGGA
ACTGAGTGTAGTATCATTGTAAATTCCAAGGTTTACAGGGGAAAACTACTCTTGTTAAATTCTAGTGTCTGATGCA
TCAGGAGGTTTTCTCATTTTCTTAATAAACTTGAGAGAAAGGTTACTATTGTAGTGTTAGTAATTTACAGATAGGAA
AATTACACATTGACTTAGATTCCTTTGGAGGCTTATGGTATACATTTGGCATGTGTTTTTCATTTGCGAAAGATGAC
TTATTTTTCAGTTATTATCTGTGGAACATCACTTGTTGTTTTTACAAAGATTAAAATACCAGCGATGATCTGCTCTCT
GCATTTAAGCACTTATTTAACTACAATGTCTGGCCGGCCAGTAGAGTTCTTTACATTGAGAACCATCCTGGAACCG
TCCTGGGGGTGACTAGGACCAAATTCCCAGCCTGCATTTTTTACACAGGTTAGTGTGTGGTTTAGCTGGATTTTGA
GAATGAAGCGTGTCTGAATGTATTGGGTGGAGGGACAGTTCTTTCCCCTAGCTCTGTCCACTGGTGCAACTCCTG
GTAAACCTTGAAACAAACTGAAAATATCCTGATCAGGAATGTTCTTGGATGATCAAAAGGAAGCGTCTTGCCAGA
CCGGCATCTGTTGCTGTTTGGTCATAGTGCACCCAGCCTGCTGACGTAAAAACAAAACAAAAGCCAGTAGGGAG
GGAGCAGAGAACTCAAAAGTACGCGTGGGGCTTTGAAATATGGTGAGTTTCTAGTTTTGGCAGATGATCATTTGA
GAAGTTTTAACCGTTAAAGTGTCCCTTCTCCCTTCCCAAAGCCCCCCGGTTTTAATGAACAATTAACTAGTATGAA
AATTGCTCTTTATTACTATTATAAATCATGGGTCATGATTATACAGAAATCCTATTGGTCTGAGCTTATTCTGAACT
GTAAAGTGTTTTTCCTTTTGTTTAACAAGTCGTGTTTCTAAGTCGTTGTCTGACTCTGCGACCCCATAGCCTTTAGC
AAGCCAGGCTTCCCTGTCCTTCACTATCTCCCGGAGTTTGCTCAAACTCCTATCCGTTGTGTCAGTGATGCCATCC
AACCATCTCATCCTCTGTCACCTTCTCCTCCTGCCTTCAGTCTTTCCCAGCATCAGGGTCTTTTCCATTGTGTTGGA
TCTTCCTGTCTGGTGGCCAAAGTATTGGAGCTTCAGCATCACTCCTTCAGTGAATATTCAGGGTTGATTTCCTTTAG
GATTGCCTAGTTTGATCTCCTTGCAGTTTAAAGGACTCTCAAGAGTCTCCTTGAGCACCACAATTCAAAAACATCA
ATTCTTTGGGGCTCGGCCTTCTTTATGGTCCAACTCGTACCTTCATTCCCAGGTGGCACTAGGTGGTAAAGAACGT
GCCTGCCAATGCACGCGGGTTTGATCCCCGGTTTGGGAAGATCCCCTGGAGGAGGGCATGGGAATCCACTCCAG
TATTCTTGCCTGGAGAATCCCATGGACAGCCTGGCGGGCTACAGTCCACAGAGTTGCCAAGAGTCAGACACGAC
TGAAGTGACTGAGGACTCAGCACTCACATTCATACATGACTTCCTTATTTACTAATAAAGTCAGTCCCAGGTAAAA
GCTTGGGTGCTGCTCACAGCAGGAGACCCAGGCATAGAAATCCAGCAACTTGACCTCCATTGGGACTGACATAT
ATATATTACTATGTATAAAATAGATAACTAATTAGAGTCTTAGAGAAGACGAACAAAGACGGGGCTACAGACTTA
TCCACTGGCATGCAAAGATACTCATAATGTCAAAAGAAAATCAAATGTAACTAAACACTATTATGGTATGATCTTA
TTTTTGTAAAAATAGAGGGGAGTGAGTGTGTTTATACTCATACACACAGAAAAGCATATAAATAAGATATACACC
AAAATATTAACACTAGTTGTAGAGGAAGGAATTACAAGATTTTAGCAAAAAGAAAATTTTAACAAATTTTATAGCT
TTTTTTTTTGCTTACCTTTATATTTAAAAAAAATAAAAGATTTTATTTTGGGGGCAGGGAGAAGTGAAGAAATTTGGA
CCTACCCAATGTCATCCAATTAAGTCAACAAATGGTTTATGCATTTTGGTAGTTTCAGTAATACAAACCAGATAAA
TTAAAAATCCAGTGGAGCTATTATTTTAGTGTTGGAAGAAATGACTGCAGTGTTAAGTGTATAAAAGGATTTAAAA
TATGCAGCAGTAACTTTTGTACACTGAAGCTAAGCTGTGGCCCATTACAGGAGTGGGGGCCACAGATGTCCAAA
ATAACAACAAAAAAATGGAATTAACATCCTACAGCTTTGGACAAAGGAAACCACTCCAGGATTATACAATGAACA
GTATGCTTACTCTTCATCCCAAACCTATTTGTATGGCTGCAAATTAACCCACCGGGGGTGGGTAGCGATATATTTA
CGGTATACCTTTTCCAAGGAATATGACTGCAGTTTCAGTTTGGAGAATGTAATGCCTTTAAGTTTTGTGGCAAGAT
TTCAGAACGTATGAGGATGAGTCTGAGGACTGCCAATCTCCCGATTTATCCCACCTCCCTTTTCCCCCTCTTGGTG
TCGTTATATTTGTTCTCAGCCCCCTCTTTAATTCTTAATACTGTGCCGTGGTAAGAATGTGCCTTCATATATTGAACC
ATTTTTTGTTACTGAGCAACGAGTTTGTTTCTAGCTTTGTGATATAAACTATATCCTCCTAGCTAAATCTGTGTGTAC
ACCCTTAGTTGATTTCCTTCACTTAGACCCTTAGAAGTTGTAATTTCTGGGTCAAAGGACTTCTATTCCTTTTTAAA
GACATTTTAATGTTTATTGCCAAATCTTCTCAAGAAACAATTGTACTAATTTATACTCTCACCAAGAATATTAGCAG
GCCCATTCTGCATCCCCGGCCACTGCAATTTTTAAATCTCTCAAAGGTTGCTTCTTCACCCAGTGGCTCAGTGGTA
AAGAATCCACCTGCAATGCTGGAGACTCAGGTTCAATCCCTGGGTTGGGAAGATCCCCTGGAGGAAGAAATGGT
AACCCATTCCAGTATTCTTGCCTGGGAAATCCCAAGGACAGGGGAGCCTGGCGGGCTACGGTTCATAGGGTCAC
AAAGAAGTGGACATGACTAAGCAACTGAACACAGCACACACTGATCTGTACAACACTGGGGTGTTACACTTTGA

ATATATATATATGTTTTTAAGTGTCGATTATTTATTTGGCTGCATGGGTCTTTGTTGTGCCCCACAGGATCCTCCGTC
TTCGTTGGCAGCATACAAGATCTTTTTAGTTGCCAGCATGTGAGAGCTAGTTGCCTGATCAGGGATTCATACCCAG
GCCCCGTGCATTGGGAGTGCCATGTCTCTATATTGACTTCTTTAACCCCACTTCAAGACAACCTAGTTGTCTCAGG
CACTGCTGTAGGCACTAAAATATTTGGCAGCTCATTTCTGGAACTGTTATTGAGTTCCCTTGAACCCAATGAAAAG
TGTTGATTTTGTGATCCTTGATGTATATTTAGGTCCTTGATGTATAGTCTGGCTAAAAATGTAATGACTGGGTGATG
AGGCCAGTACTTGGTGTTCTGAGTGCATGGATAGAATTTCATTGCATTGGTTGCAATATCTAGTGAACCAGTAGCA
CTGCTGATTAAACTGTATTGTAAGATGCATCCTGATCTCAGAGATGTTACTTGGTGGGGAGATGTCAGAGCCGGT
GGATATGTCCTTGTAACTCTACTTTTCTTGTCTCATGGCTTGAGGTAAGCATAGAGACACTTGGAGACACAGTAGT
CAGTCTGACATAAGTTTTGGTAGACAGTTAGCCAGTTATATCCCTGGAAAGAACTTTAATGGACACAGTTAAAAC
AGTAATGCCTTTCCTTAGGCATAGACCCTCCAGTGGTACTTTTCCTCCCCTCTAGATTGAGGAGGGAGCCTGGAC
CTCGGTGTAGATACTGTCATGAGGATTCCTCAGTCTGAACAAGGGACCATCAGGACTTTCTCGTCTGCCCCAGAG
GGGGTGTTTTCACCGTGATGCCCATAGCGTACCAAGAGCTACATGTGGCTGAGGAGTTGGGCTCAGGTCTCCCC
ATGTGGGTTGGGCAGTCACCATTAACCCCAGCCTACAAGGTCCCCTTAATTGTTTCCAGAACATTCCTGCAGAGC
ACACGCCCTCCACAAAGCCTACACAGATGTTCTTAGAGTGTGGCTCTAGGAAATGAGCAGATGTAATGGAAACC
TCAGTCTTGACTCATTCTGACTGTTCCATATGAGTGGAATTTGCATCTCATTTTGCATTTACTCGAACACTATTTCA
GGGCAAATTCCCATTGAGGTCATGAAATCCTGAATTTTATGCACGGGGCACCTTTCTCAAAGGAACTGGAAGCTC
TTTTGCAAAGAAAAACATGCTTTTTCCCTGAGGCTTTGATTGTGGTTGACAGATGGGGAAGGAGTTGGTGGAGAG
ACCATTGATTAGCGGTGAAAGAGGTACATCTCCCTGGAGTTCTTTCTTCCTTATCGCTGGTTCTTGTCTTGGCTGTT
CAGATAGGATGTGGAAGTGCTACAGAGAGTTCTCTTGTCTGCTCGGAAAGACTGTCAACACAGAAGACAATGGG
AAAATACCTCGAGTTCCTTTGTAAATTGACGCTATTGTGCAATATAGACAGACAGCTTAGTAATTCAGAGATCAGT
GTGGATGAAGAGGGAAACAGACTTTTAAGAAAGCAAAAGAAAGATGGGTCTTCATGAACTGGGAGGATTTAAAT
AGGTAGAAAGAATTGGGGACTGGAGCGGAGGTTCACCATCATAAAGGGTTTGGGGGTAGGAATGAATGTGGCA
TATTTCTGTGACAGCGGGGAGCCTTTTCTGGCTGTTCATTTTGAATGAATGGAGGAGTGATGATGAAGCTTGGAG
AATATTTTAATGAATTTTTATCAATCAGTTTTCATTGGTTCCCCAACCAGACTTGGTCCATAAGTGACAGGTATAAC
CCCCTGCAGAACACAATGCCCAGCTTGTCTGCTAGCTCTGAGGGTAGAGAATCCGCCTGCAATGCAATCTTCAAT
CCCTGGATTGGGAAGATCCCCAATGGCAACCCACTCCAATATTCTTGCCTGGGAAATCTCATGGACAGAGGAGC
CTGGGGGGCTACAGTCCATGGGGTCTCACAGTCAGACATAACTGAGAGACCAGCACTTTCTTCTTCCATATTTTAT
TTATTTTTTTTTTTTAACATACATGGCTTGCAAAGCCACCTAGCTGTTGAGATTAGCGTACTGGAAAAATGAAACAA
TTTCATCTTTAACTCTTCCCATCCAGCTTCCTCATTGGAGTCATTTACTCCATGAGGGCACTGGTTGATTTTCGTCTT
CATTCTGCAGTAGTGGGGCTGCTAATCAGTCATGGTTGCCCTCAGCTTTAGAAACTGAAAGTATAATTCCCCGGT
GAGGCAGTCACTCCACATTTATTAAACTCGGAATGTATTAGGCCCCGGTGATAAAGGGGGCAGCGAACTTAGAC
ATTGTCCTTGACCTTCCTGGAACTTGCAGTGTAGTGAGGCAGCATATCCTAAACAAAAGCGCCAGTAAGTATAAA
ATTATAAACAGGTCCAAGTCTCACGTCACTACTGGAAGAACCATAGCTTTGACTATATGGAGGTGTTACTTCTCAT
AAATCAGCTGGCACCTGAAAACATTTGGGGGCTTCTTGGTCAAAGCATCTGACATCTAGGGAAGTAATTTGAAAA
CTGGAAAGCATTGTCTGCCACTATTTTTTTTTTTTTTGGTTGTAGTTAGTAGGTAGGATCCTGGTTTCCGCAGCTGA
CATTTAAAGCTCTCAATGATCAGGCTCTTCATTGCATTTCCAAACTTGGCGTCTGCTGTCTGCCTGTGTGCATTCTC
TCTTCTTAACCTTCATCCCTGCATCTAGTCGGGTGATGGTGTCTTCATTCATTCTTTTTTAATAACTCTCTCTCTCTCT
TTAGTTGTTAAGTCATGTCTGACTCTTGCGACCCCATGGACTGCATGAAGGTATAATTCACAAACCTTAAAATTCA
CCCTTCTGAAGTATAGAATTCAGTCATGGTTTTTTTTTTTTTTTAATATTTATAGAGTTGTGCAACCATCACAGCTCT
AATTTTAGAACATATTTTGCTATCCCCTCTTCCTCCCAAAATAAAACCTGTGCTCATCAACCCCTCCTCCCAGCCCT
TGGCAACTATACTAACTTTTGTCTTCATGGATTTGCCTATTATGGACATTTTGGACAAATGGAATAGTACAAAATGT
GGTCTTTTGCGTCTGACTTCTTTCACTTGGCATAATGTTTGAGGTTCATCCGTGTTGTAGCATGAATTCATACTTTG
CTCCTTTTTAATGCCTGAATAATATTCCGATTGTCTGTCTGTACTACATTTTGTTAGCAGATTCATCAGTTGATAAAC
ATTTGAGTTGTTTATCAACTTCTGGGCACCCTGTTCTTAGAAAGTGACGATTCCACCTCCGGGTCCCTGACATGCT

TGTGCCTGGCCCGCTTTGTTCTGTCTTCTCCATGAATTTTCATCCCCTCCCCATTTCCAGGCTTCCCCTTGGGGCTT
TCCTACTTGTTAGCGTGTCTGGCTTTCCCCCATAGAACAATTATGTTAGTCTCTGACTTAGCACCCTGAATATGCTG
CCTAGTATTTTTACTTCTATCCTTTCATGAACATCTCCATACATTTGTTTGGATCTCAGACTCACTTGCTTGTTAAAG
ACAGTTCATTCAACAAATACAGAGTTGAGGATAAACCATTGAATAAGGCTGAGAGTCTTACCTTTGTGGGGACAT
TAGATTGTAGCAGAACTTAGGTGACTTTTTATTTAACTTTTAAAATTAATTTTTATTGGAGTATAGTTGGGTTAAAAT
GTTTTTACTGTACAGCACGGTGAATCAGCTATTCGTATACATATAATTTTGGATTTCCTTCGTAGGTGGCCTTATTA
AATACATAGATATTTTACTGCTATTCTTTTAGATTTCCACCTGACCACAAGATACCACAGTGTAGATTCTCAGTAAA
CATTACTTGATAATTATTGCATTGAATTTTCTTAACTTAAAAATCGAATGAGGCTACTTAATCCTCTCTGAACGGCA
TAGGTTGCTGTCTGGTTTCCTACTGGCACTGTAATCACAGCTTTGGAGGTGATTCCTGAAGGTGAGAGGATGGGT
GTGAAGTGGCCCCTTGGAGACTAGAAACCAGCTTGCAGAGGACAAGTCTCTCATCCTCCTCTACCCCATGTCTTG
AGTCCAGACCCTCGCCGGCGTCGTCTTATTTTGCTGGGCTGTTGGAGTCTGAAGTCTTCGTTATTCTAAGGACTGG
AATCTGGGGGAGAGAGAGGAGGGCGGGCGGAGTCTGTTCTCCACTTACTGTCCGAGATGAGACGCTTTCCTCCT
CCTTCCTGTTCATGAGGAGGAAAGCGTTTACTCGGTTCCTGATAAAGCTGAGGCTTGTCAGGCCCTGAGCAAGGC
GCCCGAGAAGGGCTGCCAGGCCAGGCTGGGAGCGCGGCTTGGGGGAGAATGGATGCATGTGCTTGTGTGGCTG
AGCCCCTTGGCTGTCCACGGGAAACTCTCACAGCATTGGTGCGTTTTTGTTTCTTTGGCTGTGCCGGGTTGTACTT
GCGGCATGTGGGATCGAGTTTCCTGACCTGAGGTTCGAAGCCGGCTGCCCTGCATTGGGAGCTCAGAGTCTTAG
CCACTGGACCAGGGAAGTCCCTCACAACATTATTAATCAGCTCTTCTCCAGTTGAAAATAAAAAGTTTAAAAAAA
AAAAAAACAAAAAACAACTTCCAGCTCCTCACGCAATGCGCCATAATTCCCTATGAGGTCAGGTGTCTGGCTTGC
CCAGCCTCTTTTGGAAATGGTGGTAGAATGGCTATCCCAGAGCCCTGTGAGGATTCAAGAGTTAACATTTGTAGA
GGCCTCTGGAAATGTTAAGTGCCTGGTGTTCTTATCTCCTTTCTGGGCATAGTTGCCTTATAAGGCTGTCCACTGG
GCCGAGGGACCTCTGCTGGGGAGCTGGGCCAGCCCGTGAGGCTAGATGACTGGCAGGCTGTGACAGTTTTCCC
AGAATGGGCTCCGATTTCTTATTTTAATACTGGTGATACCACTTTTTTTTTTTCCCCCTAATTGTTAGTGTCAGCCTC
ACAAACACTTTACCCTCAGCTTTATTACATCTATTTTTAGCCCCTCATCTCGGTGATTCTTTCCCTGTGAAAACCAA
GTATAGTAAAATCCATTTGAACAAAATAACCCCGTCAAAGTTTTTAAATTTTCTTTTTTTGGAAAGGAAGACTACTC
ATCCCAAGACGTATTTTGAAGTAAATGAATTCCTTTTTTTTTTTCTTAAGCTGTATTTGAAAAAAGAAATAGCAGCT
ATGACTTTCCCATAGCGCTCCTAAAATAATTGATGAAAATGCACAGATATTCAGTCTAGAAGACTACGAACTTTTG
TTCATTTTTCTTTTGTGAAACAGCTTTTCAAATAGCAGTATGTAAACATGGAAACTTACGTGTTTGAGAGCCGGTTT
TAAAGGTCTTCCTTCTTCCTCCGGCCATTTACTCTTGACATGTCCATTTATGCTTATCAGAACTTTCTGGAGCTTGG
CGGCACCCAGGAAGTGCTTTTGTTCAACACCCTCCTCTGAGCAGTGAGTCAGCTGAAGCCCAAGGTTGGATCTG
AAGCCCATTCCTTTTGTAAGACCGTGCTATTCCAATTGACCCCTGTGTCTCTTTCTGGGTCCCTTGATGTCAAGCA
GAAGGACTCCTGTATTTTGAGTTGGTTAACTTATAAAGTAGGAGGGATGATAGCTACTCTCAGCCTACTCCAGGA
AAGGTGCTATCAAGGTGAATGGTGTAAATAGTCCAGAATGTTGTTTTGGGATCTAGAGACAGGGAGTAGAAGGTT
AGAATATTATGTCTTAGATCCTTGTTGATCTCTGCAAGTACTGAAACCTTGGTTCAAACCTGGCTTCCTTTTTTTTTT
TTTAAATTGAACGTATCATCAGAACAACAGGTATTGTGGGGAACTTCCCTGGTGATCCAGTAGTTAAAAGACTCT
GAGCTTTCTATGTAGGGGGCATGGGTTTGATCTCTCGACAAAAAAAGAAAAATTAAAAGCTAATTAAAAAGGAG
CAACAGGTACTGAGTCATTGGTAACTATCTGTACTGGCTTAGTGGGTTACCATCCTTTTCATTAATGATACACTTTT
GCTTGCCAGGGCAGTACCTTGTGAGTAAACCTAAGTCCTATTTAGAGGCAATGAGCAAATATTTTTGGTGAGCCT
TGTACCTCCTGTATCTGTGTGAATGTTTATAGGCAATCTTGTTTATAAGAAGTGGTGCAGAGTTGGGGAACTTTGG
CCTTAGTAGTGGTGGGAAGTGGGACACAGTAGGCTGGGCCTGTTGCTGGTTAGCTCATTTGATGAGAACAGTATG
CTCATGACCCCGGGGTCAGTGGACCATCTGTGTGTAGGCCAGCCAGCTCTGCTCTGCTGGGATTCCAGGAGGCT
GGGCCATGTGTGTCACTGGTCACGAAGCTGGAGCAGATAGATCACCAGACCCAGGGAACGAATTCAAAACACA
TGCCCTAAGGATGGAAGACTGCTTGTGGCTCTTGAATTTATGAGTCTATCATAACAGTTAGACATTTTACACAGTT
AAAGTTTTCAGAGGAATAACAAAATACATCACTCTCAAGGATCCTAGGAAAAACAGAAAAGGAAGAAAATAAAA
AAAAGATCACTTTTCCCACTAGTGACAGATTTTATATTTTGGGTTTTTCTCATTCTTGACACCGTCTATCCTTGACTT

TCATGATACCATGGACTTTCACTCTCCCCCGCCGCTTTAACCTCACTCCTTTTCATCTGTTTTCCTTGGTTTGGTTCT
TAGGTGATAGTATCCCTTAGCCCTCCATTCTGCCTTTATTTCGCATTATCCTACTCTTCCATGGATGATGTCCTTATG
CCAGACTTCTCTCCTCAGCTCGATTCAAATATCTGAGTGTCCACACCACCTCCAGTTGAATGGCCCCCATACACCT
TAAACTTACCATGACCAGTAGCTGATTTCATTATCCACACCCCCCTGAACCTGCTCCCCTGTATTCAGCATCTGAG
CCACCCCTTACCTGGGTGCCCACTTCTTCCTCCTCGCTCCCTGTCTGTCTTCCAGTCCAACAGTTCATCCTCCCATG
TGTGTCTCAAGTGTGTTGACTTCTCTCCGTCTTCCCTGCTGCTATCAGGAGGGCCACTGTCAAAGCTTTCTGCACTT
CTGTGCCAGCTTTTGAAACACCTCTCTGTCTCTAGTGTTACCTCTCCATGGTCCTTGATCTTTAGGACAGCCCGGG
TTGCCTTTCAGAAATTGGAAGTATGCTCTACTATTCTGCTTAAAACCTTTGGTCACATCCTGTCACTCGGTAGCCTC
ACCCGTGACCCCGTCTCCTGCAGATTCTGCTTTTAATCAGACTGAACTTCACTGACTTCTCTAGTAGGGCACCGTC
TCATTTATTCAGGAAGCATTTTTACTGAATATCTACCGTGGCAGGTGCTGTTATAGGTTCTGGCAACATCTCCAAG
GAGAATGAGGGGGTCAGGATACACCTGGGGGAGGGCTCCCCTAAATGGCTGTAAGAGCTCTGTTTTGGGGAAA
ACCTGGGCCAGAACCGTGCAGTTCACTGCTTTCCGTGTGAGATGGCAGTGCTTGTACATTCTGTGCCTAGCACAG
TGCTTGGGATGCAGGAGGCACACAGTATTTCAGCTTGCATTTCTGCAGGACTTTGAAGGATGACTCTTCAGAAGG
ACTATATGAGAAGCACATTGTTCAGAGGAAGGGACATCTGATTTCTTCATCAAAAATTAAGTGTCTGAGGTTTGG
CAGTGTGGTTTTGTGTTAGTTTTATACTTAAAATGAGTTTGGATAGGGCTGAATTTTCATTTAAGACGGTAATTCCT
TTTTGTAACATTCCAAGTCATGTTACGTCGCAATGTGGGATTTTCAGAGTTGGGAAATTCCTGTGGAGCCAGGATA
TAGAGTGATGTTTCTGGATATACTAGTGACTTATGACTTGAAAGAGTTGCATATGTAGTAGCCATGTCGCCTGCTT
TGTCTTGGGTTTGTTTCGCATCATCCTTAACAAATAAACTAAACAAAATGATAAAGGGTAGAAAAGGCCCTGCTCT
AGTGAAGTCAGTGTGTAAATGTTAATCCTTAGGAAATACAGTATTCTTTAGTCCTACAGATCCCATTCTTTGACCCT
CATTCTGCTGCCTTCATGGTTTTTCATATATGTTTTCCTTTTTGTGGGAGAAGCTTGTGTGTCAAAAGGACAGATAC
ATCTTTTTGCCCAATCTCAGAATACAGTAAAACCTTTTGATAGTGTTTGTGATCTCTGAAAAGTTTGTTAAAATGTT
CAGATGATTTACTTTCATATAGCAACAGAGCAGAACAGATCACCTTGATTGGGCACCTAAGCGTTTACAGAATGT
TTGCAATCATTGGATGCCTTCTGTTTTCTTTCTCACTGTCTGCACTTCCCAGGGACTTTTCCTACCCAGTACCGGAG
AACTGATACTAATCAGTGACTGAAATCAACATGGAGACTCCTCAGAAAACCTGTTGTTCATGAACTTTTAGAGAG
CTGCAAAGGACCCTAAATACGATGACTCCTCATTTTTCCATTGTTAATATAATGTACAAAGTAATAAGACATGATT
TTTGTATGTGAAAATACAGAAGTACATGCGGCAAAAAAATTTCCCCCTCAACCTCCTACCCTATCCTGTACCAAAA
ATGTAATCACAGTTAATGATATAATATTTATCTTTCTTTGCACTTAGATGCAGATAAAAATGTCTTCTTACATGCAG
TACATATGATTTGGTCCCTCTCTTTATTTTAACTTGACAATAAATCTTAGATCTTGGGTATCAGAATGTACAGCCTT
ACCTCATTCTTTTCAATAGTTATATAGTATCTGTTGTATGGATGTACACTAATATATTTTACTACTTCCTGCTGACAT
TCAAGTTATTTTTCAGTGCTTTTACCATTACTAATGCTATTGCAGGAAGCATCTTTTATGCATACATCTTTGGACAG
GAGTGTGCTGGTATTTCTAACAGTAATAGAGTTGCATTTTATGCATTTAACTTATTAGAACTCAACCGTAATATGCT
CTTATGTAATTAAAGATGTAAAATTTTATTTTGCCTACATGTTCTTTATGATATATGTACATCAGTAAATAAGTTTAT
ATTTATGTATATTATGCATATTCAAACTGGTAGGTTACATACACTTGGACAACTTAAAGGATATTTAAGGATTAAAT
TTTTTTTTTTTTTTTTTTTACCGTATGTCATTTTAAAATTTACTCTTTAACTCTGGAATGTGAGTGTAATGTAAAGATTG
CTAGAATTGGATTTTCTGGGTGAAAAGGTTATATGCATTTAATTTTTTGATAATGCTGAATTTTCCTCTAAAAAGAC
TCATTAATACGCAGGTCCATCTAGTGTTCATTTCTTCACCTCCTTTTTAACATCAGATGTTATCTTTCTTTGCAGTCT
TTGGCTGGGAAAAAGGCATTTCATCACTTTAATTTGTATTTTCATTAATGAAGTTGAACATTTTCATATGTTCACTG
GTTTTCTGCTTTTAAACAACTTGGTTAGTGGTCCTCTCTTATCCAAGGTTTTGCTTCTGTGGTTTTGTTACTCACAGT
CAACTGTAGACCCAACATATTAAATGGAAAATTCCCGAAATAAACAACTCATGAGTTTTAAATCTCATGCTGTTTT
GAGTAGTGTGATGAAGTCTTGTGCCATTCCATGCTGTCCCATCCAGAAAGTGAATCATCCCTTTGTCCAGTGTATC
CATCTTGGCCATGCACCCAGCAGTTAAGATGGGAAGAAGCATATGTAGACTCTGGGTTTCAGGTGTCCACTCGA
GTCTTAGAATATATCCTCCACAAATGGTGGCTCAGACAGTAAAGAATCGACCTGCCATGCAGGAGACCTGGGTTT
GATCCCTGGTTCAGGAAGATTCCCTGGAGAAGGAAACGGCAACCCACTCCAGTATTCTTGCCTGGAAAATCCTAT
GGACAGAGGAGCCTGGGGTGGGTGGGGGGCGGACTACAGTTCACGGGATTACAAAGAGTCAGACATGACCGA

GCGACTAACACTTTCACTGTATTATAATCTTTATTTTTCTATTGTTATTGATTTCTAGGCTCTCTTTATATATTATAAA
CATTGTTTATGTACTTTCTAAAGTACTTTGGAAATGTTTCCACTAGTTTTTTGCTTGTCTTAAGAATAGGTCAAGGTG
TGATTGTTTGCCTAGAAAATCCAGAATTACCAATTGAAAAGAAAAAAAAAACCTAGAATAATAAACTTCAGTAAGG
TGGCCAAACAGGATAAGCACAAAATCACAAGACTTTTTTTTAACCTACCAGCATAGGAAAGCAGTATTAAATAAT
TCTAAATTTTAAGATACATTTTTGGCAGCAGTAAAACCAACTTGAAAGAACAACCTGAAGAATACTGTATTGTAAA
GAACACAAAAGACCCGAATAAATGGAGACAGAAACTATATTCCTGGATGTATATTGTAATGTTGCCAGTTTCCTC
CAGATTAATTTATAAATCCAATAAAAATTTCAAAGTAATTTTAAAAGAATTTGACAAACTAGTTCCAAAGTTCAATT
GGACTTAAAGATGTAAAGACAGCCAAGAAAACTTTGAAAAAGAAGAACTATGAAGGGGGACTTTCCTCACCAGA
TAGTGAAATCCATTTCAAAATGTTTTTTAAAAAATGTTTCTATGGTGCAGCATTTGAAACAGACGAGTTACTAATTG
AGGACAGTGAGGTCCAGAAAGGTGAAATGAGCAGCTGAAGGTCATAAAGCTCAATTAGGTCATTTTTCATGAAG
TAGAAAAATATTTCTCAGTTTTTGAACGGATGGCACACCATTCTAACTTTGAGCAAAACTCCATTTGGGGATTTCT
GGTTTCGTCATCTGCAAGAGTATTTTAGGAAAAGCATGCATTCAGAGAATCAACTTGCAGAACATTCACAAAGCT
TGAATAGAGTTTAGAAAAGGTGCTTGTACGCGTAAGCCAAGCCCACATGTGTTTTGTGTTGTGGTGTGTTTCCTGG
TGGAGGTGACTCAGTAGTTCAGAGGTGCAGGACTGGAAGGTGAGAGTTCTGGGGTGTATCTAGGAACAGGAAG
GTAGGAATAGTTTAGAAATTTGGAATCAGTTGACAGGTTGGTTAATATTTTCTTGATATTGAGTTAAAAGTTTCGAA
TATTGATCTGTTGCACATTATGAGAATGGCCAATTTTAATTTATTTCCCATCGATTTCCCCCTTATGCTTTAAATGTG
TGCTGTGCTGTAAAAATAAAAAGGGCATGGATGGGAAAAGGGGTGTGGAGAATTTGTAGTGACTTGTATGAAAA
TTTGGCACAAAATTTTTTTTTGACTGTGGCTGGTCAGCTGCAGAATGTTCTGTTTAAGGAACTGATAGCATCTTGG
GTGGAGCTAGGAGCAGAATCATGTCTAATCACATACTTACCAACTTCCTGGCACTAAGAAAATTATCACCAGGCA
GCAGCTACCGGGCTCATGTAACTTTAGTTCTGGACAGGCACATCTGAACTAAATCATTTTTGAAGTAACATATTTT
TGAGCAGATGCAGGCATTACTTGGCTTCCTCTTTCTGGTGATGTTTTCTTCCATCCTCTTCTGTGTTTTTGTCATTGG
TGTGTGTACGTATGGCAGTCCCATTAAAAATGCGTTAAAGTACAAATATCATATCTGAGTCCTATATTAGTGTGCT
TAAATTTGAAAGTACGGTATCACATAGGTGATCCTTTAGAAGATTTCTATGTAAATCTGTGTACCTACTATTGCAGT
GCCCTCAGGTCGCTTGTGGGCTACGTGGAAAGTATTAAATGGAGGTTGAAAGTGTTGAAAATGTTGGTTGTTCAG
TCGTGTCCGACTCTCTGCAACCCCATGGACTGTAGCCCCCCAGGCTCCTCTGTCCGTGGAATTCTCTAGTCAAGA
ATACTGGAGTGGGTTGCCATGTCCTCCTCCAGGGGATCTTCCTGACCCAGAGATCAAACCCACGTTTCATGCATT
ATAGGCAGATTCTTTACCATTTGAGCCAAACGGAGGTTAGAAAAGGTTAATTGCTGTAACAGATAAAAATAATCG
CTGGGCAGGGTATTGAGGAACGCTTGTTAGAAGCAGTAGCGTTTGGGCAGGGGTGTGTTGGACTATCTACTGTTA
CTTTATACACAAACCTGGTCTTTGTTTAGACATGAGAGTGTCCCATGGCACTTCCTTTTGTAGGAACTTAATGTACA
AGAGAAACCCAGATGTGTCTTTCTGAACAAAGTTAGACATATGAGGGTAGATTTTTGAAATCCTTCTACTTGGGCT
CAGTGCCATTATGTTCTCTTATGTAGCCTAACACACAAGAATTTGCAGAACAAATATGTTAAAAAAAAATTTAAAA
AGCCCCTTCTTAAAATGGAAAGATCCCATTCTTCATGGTATTGAATGGATGTGTGTTCATTTTCAGTTCGTATTTGT
CCACTCTTCATGCTAGAGGAACTCATTAAACAGGGATCTTTCTGTCTGCAGTAAGTGCCTTTCCAATTTAGTTGGTT
TTGAAGAGGTCATGTGTCCATTGATCCCCACTATCTAAAGTAGGGTTGAGGGGTCTTCCCTCATGGTCCAGTGGTT
AAGATATTCTCTTCCAATGCCAGGGGGCTGTGAGTTCGATCCCTGGTTGGGGAGCCTTAGCTCCCATATGCCTCC
TGGCCAAAAAACCAAACCATAAAACAAAAGTAATATTGTAACAAATTCAATAAAGACTTTAAAAAATGGTCCATA
TTAAAAAAAACAAAAAACGGAAAGTAGGGTTGAGAAGCAGGACCTGGTCCAAAGACTTCGGAGCAGCTGAAAT
GGGTATACATTGCAAAGTCCACACCCTCCTCGTTCATGGCTGTGTTCATAGCAGAGCTGTTCAACCTCACTTCTGG
CCTCTTTGCTCTTGAACAGCATCGCCATGGGATTAGCTATCTGGTGCCCCTACCTAGCCCTCCAGCTTTCTGCCCG
CCAGTGTCTTACCAGGGCTTTTAAAATTCTTACCCGGACTAGAGATCCAGACGCTCAGACATCTGTACTGTGTGTC
CCTTATCTCTTTGTTCCGTGTTTCCGTCACCTTGTGGGTATTTATAGGTCACCAGTTCTGCTTTTGTTGGAAGCCCTG
GTGGCAGGGCTTGGCATCTCTTTCCTCTTTGTGTACGTGGGGTCTGGTTGCCCAGCTACCTTGGAGGGAGAGTGA
GGAAGGGTCTAGAAAGGGAAGCCTGGAGTCTGGGTTAGACTCTGACTCTCCTTGTTCTCTAGGGTGGGCGGAGC
TCCAAGGGCCCAGGGAAAATCTATTGCTGGGGAAACGGCCACTTCCCTTCCATTTCTTCTTTATGGTCTTTCCTTC

TTTTTCTTCTCTTTTTAGTTCTTCATCTCTGCTTATATCTCAGACCCACTGGTGCTCATCGGGACCCAAGAATCTGCA
GAGTCGATCGGGTTGGGGGTGGGAACTTGCTGAAAGAATAAGGTTTAACTTTTCTGTGAGCAGACTCAGGGATC
ACTTGAGAAGAAAGTTTTCGGGATGGTGGAGAGTTTTACTCTGGGTCTGCGTTCAGAAAATGTAGATGACCTTTC
AACTTCTTCAGAGTGCCTTGAGATTTGGCATGCCAGTGTAGGTTTTGAATCCCGGTCTCCTCTGCTGGCATTGTCA
GTTAATAGCTGTGTTCCTTTGGGCAAGTTACTTGATTTCTCTGAGGCTGTGTCTTCCTGTGTAAATTGCCAGTCACA
AAATCTACCACATCTGGGGCTGCATGTGGGCTTCCCTGGTGGCTCAGATGGTAAAGAGTCTGCCTGCAATGAGG
GAGACATGGGTTCGATCCCTGGGTCGGGAAGATCCCCTGGAGCAGGGAATGACAACCCACTCCTGTATTCTTAC
CTGGAGAATGCCATGGACAGAGGAGCCTGGTGGGTCATGGTCTATGGAGTCCCAAAGAGTCTGACATGACTGG
GCAACTAATACATATGGATTACAGCAATAGTAATAGAGAGTATGCCTCCTTGGATTATAAAGGTGTGTTCTACAA
GTACAAAGCTACCATTATTATTACTACCTTTATTATTATTTATTTGGAAGGGAGTTATCCTGGAGAAATGGATGAG
GACAGGTTATGGAGAAATCAACCAATAAGCCTGGTTTTTGTGACATAGAACATTTAATTATAAATCACTTACTTCA
GGGGACTTTTCTGATGGTCCAGTGGCTAAGACTCCATGCTCCCAATGTAGGGGGCCTGAGTTTGATCCCTGGTCA
GGGTGGATTCCACATACCGCAACAAAGACCTGGGACAGCCAAAGAAAGAAATACTAAACGTGTTTTTTAAAGAA
AATAAAATCACTTTAGGTGCAGTTATGTTGGCTTAATAACTAGGTTCATAATCTGTTTTGTCTGCTAGCTAGGTAAG
CTTTAACCTTTCTAAAGCCATAGTTTCTTCATCTGTTTTTAAGAAAATGATAATATCTACTTCATGGAACTGTAAAGT
AACCTTGTATGTAAAACATGTGGCCGAGTGTCTGACTCAGAGCTTGACCTAAAGGATCTTATTTTTAATTCTTAGT
AAACTTTGAATGAGCAATAAATTCTATAGTTAAATTATATCAGCGTATTCTCTGTTCCTGGAAACATGGCTGTTTGC
AAAATTTTTGACACTCTTGTGTTATTTTCCTGGAGAAAATATACATACTCAACAATAGCATGAGCCCATTATTAAGG
AACACTCCTCCATAAAACCCCTTTTAAATTTTTTGGGCATATCATGTGGCGTGTGTAATCTGTTTCCTGACCAAGGA
TTGAACCCATGTCGTCTGCATTGAAACCATGAAGTCTCAACCACCAGACCACCGGGAAGTCCTTACTCTGCTCCA
TTATTACTTGGGTAGAAATTTGTCTTCCTTCAAAAGTTAAGCCCAGCTCTCCAGTGTGTGGCAGTGGATGTAAGCA
TGGATACTCCTCTCAGCATTCTCTGCTCCCGTGATATAAACATGTATATGTAGAATAAATTTTTTTTAACCACCAAC
ATCTAGCATCGTGAATAACACGTGGTAGTAGCTTAGTAAATGTCTCTTTAATTTGGTTTGATTTTCTGACTTTTAAAT
GTTTTTTTAAAAAAAATATTTTTTGTTTCATGGAGTTATAGTATTTGCACTTGTAGTGGTTATCTTGGTGTTTTGTTTC
ACACTGAACAGGAGGCATATTACTCTGGATATTGGCATGTTACTGAACATGACTTTTTTTCTCAGTTTTGTTGAAAC
AGCTCCTTTCCTTAACCAAGGAATTTTGGTGAGATTTCGCGTATTTGTCAAACACTAGCTTCCAGACACTGTTCTAA
ACATCTTGCGAGCCTTACCTTACTGACTTCTCACAACAATCCTATGAGGGAGTTAAAACCGTGATCCCCATTTCAT
ACTTGGAGAAAGCACAGAGAGGGGAGGTAACTTGCCGGAGCTCACACAACTCTGTGATCAGACTGGCAGTGAA
CTTGGACCACCGCTCTGCCGTTTGCTGCTGGGCATGCTGCATGCCCAAAGGCTCGTCTCTGTGTCTTTCATGAAAA
GTCGCTTCTAATCCCGTGGCTTTGTGCAGCAAAGAAATGGAACAGAAATAGAAAAGATGCTGACAACAGAGGAA
TTGTGCTATGTTGCAATGCTGGTGATATGATGTAAGATGTGATGCTGATGACATGCTGGGTTAGCAATGTTCACAT
TCCATCAGGGGACCCAGTCGCCAATCTGCGTATAAGCTTTCTTGTGTTCAGACTCTTTGGTGATTATTATTCCTCAG
AGGGCAGTCATCCTTTTAATCTTGTGGTAGAAAATTAAGAGCGAGTCTCACTGAGGTATGGTTTATTTTTAAGTTTT
CTTTTAAATATTAGTTTTGAGAAGTTAATTAAGTACCCAGCCTGTGAATCCAGCGTCTTGGATTTCCTCCGTGTCTT
CCCCATTTAGAGGGCAGGTTTTCTTAGAGTTAGATTACAGATTGGTGAGTTTATGCCTTATCAGTAAATCATGAAC
ATTTGGGGTCCTGAATAGGTGGCCGGCTTGCATAATATGACCTGAGTAAAGTTAGGCAAACATAACTATCTCAGA
TCTTAAAGGCATTTTGAACAGAGCCTTAAGCTAACGTGTTTGTCAGCTTTTCAAGTAAAGCTCAAGAGGCACGGA
TACTTTCTCATTTTGCTTGCACTGATTTCTTTTTAAACTTATTATGTCTTAGGGTTGCCCCTGGTGTTAAAAGTTACG
GGGAGGTCTGGTCTGCTTGAAAGCAGTGATTGTTGATTTGAGGGGTCATTGATCCCTTTGAGAGTCTGGTGAAAC
CTGTGGATATTCTCCCTGGAAAACACACACGCAGTTTTGTTCCTAACCCCCTAAGGGCCACAGACCTAGACTTTA
GAGTTTATGGAAATCCGTCATGGGAGCCAGTCACCCTCCCCTTCTATGTGCCATCTTCTGCCTGCCCGGCTCTAAA
AATTTCAGCTAACAGGTTGGCTTATCACACTTACATTCTTGCTTGGACCCCAGGACTGTTTGACTCTGGGGAAGTG
TTATCTCAGAAGGGAAGAAATGATACTCCTTTAAACTCCACCAGATATTACCCCTGTAGGACAGGGCTCCCAAAC
CTCCGGGATCTAATGCCTGGTGCCGCAAAGGTTGGGGGCGGTTGCTGTAGGAGACAGCTGTCAGTGGGCCCTTT

CCTAGCCACCATTAGTCAGGTGGGGTGAGGCAGAAAGGGGCCCAGTGAGCGAGCCTTCTCTCCCCTCCTGCCCC
ACTGGATTCCGTGGGTTCGCTCACCTTTATGTACCTTGGCACATGTTGTTCTCCACCTGGAATGCCCTTCCATTCTT
CCTGCTCCCCAGGAATGCGTGCTCATCTTTCAATACCCAGCTCAAATGGAAGCCTGGCTGAACTCCCAGGCAGC
CTCTCTCTCTCTTTGCTGTGTTGCCACAGCAATGTTTAAAGGCCTGGGTTACAGCGCACATACTGTGTTGTAATTAT
TTCTTAAAAGTTTGGCAGGCTCCACCACCTGTAAACCAAGCACTTGTCTTAATTATCTTGATCACTCGGAACAGTG
TTGGATGCTTGGTAGGTTTGCCATCCAGCGTTTAGACAGGTAGGCCGTGGGGTCAGGAAGGCGGAGTGCAGATT
CTGCCTTCATGACCTTGTGAGGAAATGACTTCTCTCTGCCTCTGTTTCCTCATCTGTGGGCTTCTGGGGGCAGGTG
GATGCCTGTTGTCTCTGGCACATGGTAAACTAGTCAGCAAGTGCTAACCATCATGATTAATCATAATATTAAGCAG
TTATATTATTGAGTGAAAAGCTCTTAAGTAATCCAGCTGTAATTGGAGATAGCATTGTTTAAAGAAATGTAGTCAG
TGCTGTACTGTCCAGCCAATCTAGCTTTTATAAGGCACCCCAAGAGGCTTGGTAGGACCCCCTACTTTCAGCCTAT
TATCCATCTCTGTATTAGTATTAACTGTGAATTATGTGAGGGCTTTAAAACAGTTGGAAGTAGGTACGTCCTGTGG
GAGACTTAGAAGAATAAACGCGTGGTTACCAGAGAGAAGGAGCAGGGGAGATAGATGGGGAGTTTGGAACTGA
CATGTATACATTACCACATTTAAAATAGATAACCAACCAGGGCCTACTGTATATAGCGGACTCAGCTCAGTATTCT
CAAATAACCGAAATGGGAAAAGACTTTGAAAAAGAATGGATATTTCTGTGGATATAACTGAATCACTTTGCTGTA
CACCTGTAGCTAACATACCACTATTAAGTAGCCATACTCCAATATAAAATAAAAAAATTAAAATAAGTACTGTGGG
GATATAAGACTTTCTTTTCTTTACCTAAAGAAAACGCACACAGTTTTAAGGTGTAGTTTAAATGTGTTCACAACCTT
GTGTTTTTCACATCACTTTCAGAGCATCACTAGCGATCTTTGGGAAAATGGAACAGGTCACCACCATGTGTAGCCT
CAGCAGTAACATTCGGCCCTGTGGGAATCCCTAAGAAACACTTAATCACAAAAGCATTTGCTTCTGCTTCTAAGA
CAAGTCAAAGGTGGTGAGAAGAAAACATCTTCGAAGTTTTTTCCTGTGTGTGTTTTGTTTGCAGGCAAGCTAGAG
ACATCCTGCTAAAGTCATAAGTGTCATAATCCTGCTTTAGAACTGGAAGGAGCATCACTGGTGCAGCCTTTTCTCC
TTTTTCAAATCAGGAGACCAAGGTCCAGAGAGTGGCTTGATTTGCTGTGTGACATTGTGTCTGAAGGTGGGATGG
CTCTATGCCGCCGGTCCTCACGTGAGTCACGTGGATGGCTGTGTGACCGGGTCGGGGTGAGAGGGTGCTGGAG
AGCCCATGGTCTCTGCGCAGGACGAGAGCATCTGCCTTCCAGCATGCGCTTGCTTCTGGAAAGCCTTCCTGGACT
CCCCAGGCAGTATCTCCTTTATGCTCGCAAAACTAAACTTTGTCTAAATTAAAAGCCGTGAAATCTAGACCTCCTG
GAAGGGTATTTACTTTTCCCTTTAAAAAAAAAGTAAAACAATTAAGCTAATGACAGTGGTGGAAGCCTGAACTAC
ATCATTCAGTGACCGATATATATGTCCCCCAATTTTTGTAGGGGAATATAAGAGTCTATAATCTTACTTTTCAGTCA
AAGTTTAATCAATTATAGTAGGTCTCAATACATGCTTATTAAGGTTTCTTTTTTGGGGGGCGGGTAGGGAGTCCTT
AGGTCACCCAGCAAGATCCCTTTTGTCAATTTCTTTATTTTTAATTGGAGGATGATTGCTTTACAGTGCTGCATTGG
TTTCTGCCATACAACATGGCGAATCGGTCACATACACACATATACACACACACCCTCCTTCTCCTCTTTCATCAGT
TTCTTGCTTCTCTTTTTCATGTTTTGGCTTCTTGGTGTCTACATAGTTTATAAGCTCACCAGCCCAATAAATGGTACT
ATTATCTCCAGATTTCCATGCTTTACAAAAAAAAAGTATATACTTGCTATTGTCTCCTTTTTCAAAGTGTGGGTCAG
TTTGTGAATTTTAAACTATTTTTCGCATGAAAGAAGGGAAGGAGGAAGTGCTGTAGAAGTAATTAAATGCTAGTG
GTTTATAAATTAAATTCCTGAGCCCTAGTAGCTTGACTTACAGAAGGTTCCTTTTGTTGTAACTATATGGTTATATA
ATCTACACTTAGCAAATTTTGAAAGTGTGAAATCTCACAATTCCATCTGCCACTGTTGGCAGCACAGGAGAGAAC
CATACTACTTGTTCTAAGCTTGTAGTATTTCTCTAAGAAACAATAAGTTGCATGTCTTGATTCTCTGCTTCATGTAG
AATCAGGATATTTGAGGGGTGAAAACAAAGAAGAGACTTTCTCATGCATATCCCTGAAATGTACTTTTTGGACAC
GTTGTTCAGAATCTAATAACTAGTGAGTTGTTGAATAACCTTGATCCATTCCTCCACTGTATGTTACAAAGTAAATA
GCATATCAAAGAACAGAAATGCAATCACGACAGCATTTAAAAAATTAGTGGTATTGAAGTGTTACATGGAGAAA
CCAGTGTTTTGAGTTCCTGGTAGCTATCTCAGTTACTGATTGGAAAAAGTGGCATAATCATGCAGTAATCCTTTCA
TATGTGTTTGAGAATATATGGGTAGCTTTGAGAGACATGTACTAACACTCTTTATTTTTTATGGGATACTGGGCTTT
TTATTTAATTGGAGGATAATTGCTTTACAATGTTCTGTTGGTTTCTACCATACGACAACGCAAAGCCTCGATAAGAT
ACATATATCCCTTCCCTCTTGAGTAATAATACCTTATGTAGTAGTTTGTCCTAAAGGGCAAAATTTTAGCTTCTTGC
TTCCTCTTCATTTACTTTGCTTGAGCTATGGATGAACATGCTGTGCATTTTTTTGAAAACCCATCTCTTATAATTCAC
TTATTCCGATAAGATGTATTAAGTGTCTCCCATGTACTTGGCACTGTACGAGCTGCTGAAACTTAGACAAAAACAA

GGCCATTTTCTCAGTGAAGGTGATGAGAAGTGAAGCAGGTGACAATGTAGGAAGATAAATAATTACATGTGGGT
ATGATTTTCTATATAGTAAAAATGGTTTTAAAGTGAAAAAGGCCTACTCCATTCACTCTCTTCTCTAGTTGAATTAG
AGTGCTTATCAGTATATGAATGACTGATCATACAGTTTTGTTGTCAAGAAGACACTTTTAAAGGCTTCTGTGGTGG
GTCAGTGGTAAAGAATCCGCCTGTCAACACAGGTTCAATCCCTGGTCCTGGAAGGTCCCACGTGCTGCAGGGCA
GCTAAGCCATGCGCCCCAGCTGTCGAGCCTACGCTCTGGAGCCCGGGAGCCACAGCAGAAGCCACTGCATTTA
GATGCTTCTGCACCACAACTACAGAAAAGCCTGCGAAGCAACGAAGACCAGTACAGCCAAAAATAATAAAAAA
GGAGAAGACTTTAAATTTTTGGTTCAACCTCTCAAAGATCACTTTTCTTGAGAACCAATCTTTAATTTGATTTACTAT
CTGATAGTTGTTTAAAGTGACTCTTTGGTCGAAAAGTTATGATTTTCCGTCCCTTTGAGATGTCCGGAGTCTTTGGA
TAGATGGAAAATTGCAGTCAAGTCAGCAACCCTGCATCTCATTTACATTATTTATTTATTCTTTTACTCTTAAGCAA
GGCATCTTCCACCAGGACCTCTGATCTGGCACTGAGAAGTCCTGACCGAGAGGTGCTCGCGACGTCTTTTGATCC
TTGACGATGTTGGTGCCAAGGGCTATTTAAATCCTCCTTGGAGTGATTTTTTTTATCCCTTGAGGACTATTGCCTTC
ACTTAATGTCGCTTACCTGTTTTGCCTTCCTGTCAACCAAGCCACTTTCTTTGTTCCTTTGGCTGCAGTGACTATCTC
CATGAAGGTAGTTATTTTAAAAGCAGATGACAATATTAAAATCAGTTCAGTTCAGTCACTCAGTCGTGTCCAACTC
TTTGCGACCCCATGAATCACAGCACGCCCGGCCTCCCTGTCCATCACCAACTCCCGGAGTTCACTCAGACTCATG
TCTATCGAGTCAGTGATGCCATCCAGCCATCTCATCCTCTGTCGTCCCCTTCTCCTCCTGCCCCCAATCCTTCCAG
CATCAGAGTCTTTTCCAGTCAGTCAACTCTTCGCATGAGGTGGCCAAAGTACTGGAGTTTCAGCTTCAGCATCATT
CCTTCCAAAGGGTTGATCTCCTTCAGAATGGACTGGTTGGATCTCCTTGAAGTCCAAGGGACTCTCAAGAGTCTT
CTCCAACACCACAGTTCAAAAGCATCAATTCTTCGGCGCTCAGTTTTCTTCACCATCCAACTCTCACATCCATACA
TGACCACGGGAAAAACCATAGCCTTGACTAGATGGACCTTTGTTGGCAAAGGAATGTCTCTGCTTTTGAATCTGC
TATCTAGGTTGGTCATAACTTTTCTTCCAAGGAATAAGCGTCTTTTAATTTCATGGCTGCAGTCACCATCTGCAGTG
ATTTTGGAGCCCCCAAAAAATAAAATCAGATAGACTTCATTAAATATAGCATGAGAGTATGGTATGCCTATTAGTC
ATATGGCTAATAGTTTAATAGTAGCTATTGTAATAAAACCAAGTACAGCTTCTTGGCTCATTCGTAACTTTGGTAGT
TTCAATTATTTGTTACTATTTATAGCTTTATTCTTGATAGAACAAGGCAAGGAATAAATAAATGGGAAAATATCCTT
ACACAGTTGCTTTCTATCTGGCTATATTGCAATGCAGGAAAACAACAAAAGCACAACTCTTGAGCTCGGACATTC
CATTGACTCAAGAAAAATCACCCAAGCTCTGAAGACAGCTTTTATTTGGAGAGCTAGACTTTCTGAGTTTTCCTGT
GTGGAATGTTCAATAGAAATCCAGTGCAAGCTTCTGCTCCTCTAAAATGATGATGACTTTTGCCTAAGAATAGACC
CTTCTTTTAGTATAGAAACCAGATAAGCTGTCCCACAGTGATCCAAACAGCTAGCAGCCTCACCCCCACCCCACC
CCCAAACTTGAATAACAACACAGCAATATAATTTTACCGTTTAAAACTTGGCTGACCCAAACATGAACTTTCAAAG
AAAACAAGAGTCATTACTAAAAGAGATTCCATTTCTTTCCGGGGCCTAATTTAAGATGACTTTCCAGAACTTTGAA
AAAACTTATTCTTCCAATGGCCACTTAATTGTGAAACAAAATAAACACCCACAAGGGAGGCAAAAGAGAGAGTT
CTCTTATGTTTGGATTTTAAAATTATCCTTTTTTGCGGGGTGAGCTTTTTCTTTCTAGGGTTGTCATCCGTTGTTTGG
AATGATTTATCAGGTTCCATCCGAAGATTATACTCTTCTGTGAATTTTAAGGGTGATGATTTATGAGTAACTCTTAG
TAAGACTTCATTTCTCAAAATTCTGCCCTGTTAAGCGTCTCATAATACCTCTCTAATCTGTGTGCCCTGGGTTATTC
AGAACCAGTGAGTGATGGTGTCAGGAGTCTTAAGCCTAGTGGGCCTGGCTTCTAAGCCCTTGCTTGTTTTAGTGA
ACCATATTGCTTTTTATTGGGTATGCCTTTCTGAACATTTGGAAACTTCCTAGAGATCTGAAGAATTCCTTGGACAG
AGGAGCCTGGTGGGCTATAGTCCATGGGGTCGCAAAGAGTCGGACTTAACTAAGCAACTAACACTTTCATTTTTC
TTTTACTTGTGAGAATTGAAACAGCTTTGGGGAACTAAAAGAGAGCAGTCAAAGTAGCACAGCAGAGAGTGGAC
GAGCTTTGAAGTCAGGCTGGGGTTTAAATCCAGACTCTGCATTTTGAGACATTGTCTTTGGGTAGAATGAGTCTCT
TTGGCCTCATCGGTCCTTCATTTGCTGTAGCAGGGTCATTGGGATGATGGCATGATTGTATGTATAGGCTAGAATC
AACTAAGATACAGGAGGAGTTTATGACATGATAGCTGTTTTACCATTGTTGATTCGTTATTGTAGTTGTTGACTGG
ACCTAGAACTCCTGTGTTCTGATTTTTGCCATTGTGCACATAAGACCTGTCACTGTACCTACCCATGTTGTCTAAGT
GGTGATGACTTCCAAGTGTGAGTTCCTTGAAGAATAAGATAACTGAGCAGTCATGAGTCTCAGGTGGTCCTAAAC
CAATGCAAAACTTCCATTAGGTGATTGAACCTGTATTTTATTCACGGTTTGAAAAGAAAAAGTGAGAAAACTGTTC
AGAATCGTTCATCAAGTCATTTTACTTTTCTAAGCAGTAAAATGAGGGAATTGCATTAGATGGTCTCTGAGTTACC

TTCTGAGTCCGAAATTCTGTGATTGGCATTAGACCCAGTAATAGGATCCTGGGACCTTTTAATTTCCACAGTTTGT
AGAAAATTAGATGTATCAAGGATTATTACATGAGCATTGGAGGTAAGGGAGTTACTATCAGTGGTCTTTTGTGTCC
ACAAGAACAGAAAAAGGAAAAAGTCTGATTTTACCAGATTCTCAAAAATAGTGAGTACCCCCTCTTCTCACACAC
ACGCACACTAGTACACACGCACTAGTACACACGCACTAGTACATGATTTAGTTCTCCAAGTGTAAGTTTTTTTCTA
CTTGTGAGCAGTTTATAGAATTTCATTTAACCAGCTACTAAGGTCACTTGTGGAACTGAGAAAGGAAGACCTCTA
CATCCTTAGGTAATTTATAATCTTAAGCGATGCTGAACTATATAAAACGACAATGATTCTAGAACATGTTTCTAAG
GGACAGATGTTCAAAGAAGACACATATCCATATGATCCAAGTGGTTCCTAACTCCTTGGATGCCAAGGTAGAACC
CATACCCTCTGGTCACCTTTTTAGGTGTATTACTTTTTTTAAATTAATTTTTTATTGAAGTGTAATTGATCTGTGATAT
TCTGTTATCTTCAGGTATATAGCACAGTGATTGTTTTCTATATATATACGTATATATGTGTATATATATATACACATA
TTTATTTACATACATTTTCTTTTCCAGATCCTGTTCCCATGTAGGTTATTACAAAATACTGAATGCAGTTTCCTCTGC
TAGACAGGAGGTCCTTGTTGTTCATTTGTTGTTATACATAAAAATCCCATGGACGGAGGAGCCGGGTAGGCTGCA
GTCCATGGGGTCGCTAAGAGTCGGGCACGACTTCACTTTGACTTTCACTTTTCACTTTCATGCATTGGAGAAGGAA
ATGGCAACCCACTCCAGTGTTCTTGCCTGGAGAATCCCAGGAACAGAGGAGTCTAGTGGGCTGCCGTCTATGGG
GTTGCACAGAGTCGGACACGACTGAAGCTACTTAGCAGCAGCAGCGTGTATATATTACTCCCAAATTCCCAATTT
ACCCACCTCCCCCCACCCTTCCCTTACGGTAACCATAAGTTTGTTTTCTCTGTCTGTGAGCCTGTTTTGTAAATAGG
TTCCTTTATATCATTTTTTAGAGTCCACATATAAGTGATACCTTAGGATACTTCTTTTTCCGCTTTGCTTCACTTAGTG
TGATAATCTCTAGATCCGTTCATGTTTCTGCAAATGGCATTATTTCATTCTTTTTTTTCATGGCTGAGTAGTAGTTCG
TTGTGGGTATAGACCACATCTTCATCTGTTCCTCCATTGATGGACATTTAGGTTGCTTCTGTGTCTTGGGTATTGTA
AACAGAGCTTCAGTGAACATTGGGGTGTATGTATCTTTTTGAATTATGGTTTTCTCTGGTTATAAGGTGCATTGCGT
TTTGATTTAGTTCTTTTCATACATTTCATAGATTTTTGTAATTCGGTAACATGTAATTCATTATGGTACATGACTTTCT
AAAGTCAGAGTCTTAAGCTCTAACTTAGAATTCTGTTCATGTCAGTAATCCTTATGGGGTAATGAGTAGTATGTTG
GGTGATATACACAGAAAACCATGAAAGCTTAAAGAGATAGCAGCCAGTGAGGATGTGTGCACAGTCTGTGTGTG
CGTGTGGTGATGGGGTGATGAGTTATGGAAGTCTCTATCAGAAATGATCTTGAAGAGGTTTCAAAGGTAAGTTGG
TGACTGGGAGAAAAAGAAACAATTTTTGGTTAGATTATTTAAATCAGTTATATACTGATGCAAGATGGAAAAATTT
GGAATATGGATAAATACAGGAAAGGAACCAATGTTTCTGACATCTCCTTAAGTCCTTATTTGGAATTTGTTTCCAA
TAATAATCCCCCTTTTTCTTTTGAAGAAAATATCTGTTCATAGTAAAACAGACTAGGAAAATAGAAAGCTTCTGGT
CCCAGAGAATGTTTAGAGATTCAGAAAAGTAATGACTCCAGGCTTTTCATTTTTCTGAATCCTTTAAATGTTCCTAC
TGCCAGGAGCTTGCTCTTCTTCAGGGTATTCCGTTTGACTATAGACACTTCAGTGGTCTACACACCTGGAACTGTG
ACTTCCATGCTTGATGCTATTCCTGCCTAGTTGGATGCTGACATAGTTAACCTACTCCTTGTATAGGGGCTTCCCA
GGTGGCGCCAGTGGTAAAGAATCTGCTGCCAGTGTAGGAGACACAAAGGGATGTGGGTTTGATACTTGGGTCCG
GAAGATCTCCTGGAGGAGGAAATGACAACCCACTCCAGTATTCTTGCCTGGAAAATCGCATGAACAGAGGACCC
TGGTGAGCTACAGTCCCATGGAGTCACAAAAAGTTGGACATGACTGAATGTGCGTGTGCATTTGGTTGAACAGG
GCAGCTCTAAAAATATTTTAAATGTGCTATCAAGTTTCTCCCAAGCCCTTGTATTTTTCCCTTTAGGATAAACATAT
CTACTTTGATCAACCATTTTTATTTGCCATAGTTTCCAGTTCCTTTCCTGACTGGATTGCTCTTCTGGAGAGTCTTCA
GATGGGATTCTGTCCTCTGAGCACAGCCCTTCTATTCAGGATAGGTTGACACCATTTTCATTCCTTTGTCTAACAA
CATTATTGTCGCTCAGGACTACACTGGTTTTTCTTGGCACATATTGGTCGGCCTGTTTGGCTTTTTCCCCCAAGACT
GGGGCGTGTGGTTTGTCGCAGCTTGCTGGTGCCCTTATATTTTTTCATGCAGTTTTGGTTTGCCTGTGTATTTGTTTT
CAAAAGAAAGTTGGATTCACTGTGTTGAATTCATATGATTGGATTTCGTACTAGCAGAGATCCTTGTTCATGGATT
TCATGCTGCTTGAACATTCGCATAAGAACAATTCGAAGTGAATAAGTCATGAACCTTTGTATGTTTATATTCCTCGT
CATGATATGTGTAGCAATTTGACCTTAAGTTGTTGGGATTATAGGTCAAACGTTAAAATTGATCTTATCAAAGCCT
GCCATTTGGATAAACAAGTTCACTTCAGAAGAATTTTGTAGTGAACTCTGAGTGCTTAAAAAAAGAAAAAAAAAA
CCACTGTTGTTTTTACTTAGGAAATGATTTATCTATTATGGAAGTTTGTCTTAAAGCATAATTTTAGATATTTGCTTA
GTGTTCATTCCGATCTTCCTCTTCCCTCGCTGATGAATTAGCCTCCTGGCTTTTAGTAAGATAACAGCAAATCAAC
AGGGTTCTCTGCATATCTATTGTAAAATTACTGAGCCAACTGATCCTCTTACTTCCCAGGTTTTAGGATGGTCATTG

TAACGTTTATCAGATACAGAGTATAACTGGGTAGGTTCGCCACTTCCGTCCTGTTTGTGCCACAGACTGACACTGT
GTAGAACCAGGTGTCGATGGTGATCATTATTACAAGGGGTAAGGAAGACGATAGCAGCCCGGAATACCATTTTC
CCTGAAACTTCTAAGTGACTTGGTAGAGGAAATACTTGTTCAAGCAAAGGTGTAACTGATTACCTTTCACAGAGG
AATAAGAATTATCATTAAGTTCTACATTTGGAATTGAATTATTCAGTCCTTTATCCTTACACACGTGAGAAGGTGAA
AGGCATTCAAGTGCAGACTATACTTCTCTACCTTTTTATGTTTTTCTTTTCCTTTGCATTAATGATATATATTATTAAA
ATATCCTGGATGTGCCATGATTTACATTAAGTTTGTATTTATTACATACAGAGCTAGAAATAGAAAGACATGGTCT
TATTTGAAATATCTGTTTGCAGAGGGGAGGTATCTGAATTTACTTGAAAATGTCAAAAATGCTCTGATACTCTAAT
ACTGGTGGTTATTTTGACCTTATTTTAGACAAAGTTAATGTGAGCTGTGATCAGTCACTACTGTGAAATATTGTTTG
GAGGATAAACTTTGTGTGCGTGTCACTGAAAAGTAAATCTCTTTAATCCTAAAGCCAAATGGAAAGGATATGTTCT
TTCTTCGGCGTTTCCTGTTGGGTCTTACAGGAAGAAGTTGAAGGAGTCATGAATTGGCACTTTCCAAGAACTTAAA
AAGTAATTTTGATAATTCATAGAGCAAAATCTCCTTGGAGAGCCCTTTTCCAGGATAGCCTAATGACTTGGAGACA
CTGATTCACAGTTTACAAGGACCCTTCTGGAACCTAAGAGCCCTTTAAATAGTCTGGTTTGCAGCGAACGTCTCA
AAAAAGGAATGTTGTGAAAAATCTTTTGACGTTCTGAAAGCGAACAAGACAGAAGTTCGTCTTTAGAGAGTTAAA
CTTCCATTGGTTTCTCTCTTGCCAATTGTTTCTTTTATATCATCAAAAATTTTTAATCGTGGTTAAAAGATAAAATTT
GCCGTTTTTTTAAGTGTACAGTTCAGTAATGTTGAGTATATTCACATTGTTGTGCAATAGGTCTTCAGAACATTTTTT
TCCTAACAAACATGAAACTCTGTTTCTTTGCATTCCCTGCCCCCCACCCCAGTCCCTGGCAGCCGTCATCTACTCT
CTGTTTCTCTATATTTGTCTGCTTCAAATAACTGTGTCTAAGTGGAGTCACACTATGCCTTTCTGTGACTGGCTTGTT
TCGCTTGGCATCATGTCCTCAGGTTCATCCATACTGTAGCATGGGTCACAATTTCCTTCCTTTTCAAGGCTGAATA
ATATTCCATTGTATGTATAGACCACATTTTGTGTGGTCTCTCTATCGGTAGGCATTTGGGTTGCTTCTAGCTCCAGG
ATATGGATTATGCTGCGGTGAATGTGGGTTCGCGAATACCTTTATAAAATCCAGTTTTCAATTCTCCGGGATATAT
ACCCTGGGATAGGATAGCTGAATCATAACGGTAATTCTATGTTTAATTTTTTGAGGACCTGCCATACTGTTTTCCTT
AGCATTTACACCAATTTGCATTCCCCACTAATGGTGAAGAAGCATTCCAGGTTCTTCACATTTTCAGCAACACTTT
GGTGTTAGGTTCTTTTCTATAGTAATTATCAGTTTTTCACATTAACCATAATGAACAAATACTTTGCGTGTCTGAGG
GTACACAAATAGGAGATTTGTGTGTAGTCTGAAAAGCAGGGGTTTGGATTGTTCTTTAACTTGCTTTTAATAGGTC
GAGTGCACTGTTTCCGTCTTGATTTTCAGCAAAGCCAGTAGGGGAATTTTTAACCAAGGTAGAAAAAGTGGTGGA
ACATCATGGTAACTAAAGTTGCCGATTTCAAACTTGGCACAACTATTTATGCTGATAGAAATAAGATAAAGTTCAG
TCGACCAGATTGTAGGGGAAGCATTATTCATGTCACAAGAGTTTACAAATAACAGGTGTACGTAGCGCACCAGC
GTCTTTCTTGCCCATTAATGGAAATGTTCCAGAGACGGAGCAGTCTAGAAAGATGGATAAGGTGGTGGATCTCTT
CAACTCCCCAGGGTTTCACTTTTTCCATCATAATGCTGCTTTTCTGTCCTTTCTCTGTGATGGTAGAGGCTAGGTAG
TATCAGGATTGCTTCCTTGCTGTTATGCCACAACACTAATCTGGAGCAAAGGGCTGTGTGAATGTTGCTTGTTTAT
GAAATACAGGCATAAAAAAAAAAAATCAAATACAGGCATAAACATAGGCCCGGTCTGTTTCCTTAAAATACAGTA
CTTATTGTACTTTTCCCGAAGCTTAAATATGGTTTTTAAGAATTTTTTTTTCAGTGTCCCGCATGAATAACTTTGGTG
ATTATGATTAGAAAACCCCCAACCTCATTCATCTGGATGCTGTAAGAAAGAAAAGACTGCATAGCATAATGGTTA
AGAGTTCAGGCTCTGAACTGCTTGAGTTGAAATCTCAACCTTTAACAACTTTGTGAGCTTGGGTAGTTTCCTTGAT
CCTTAGCCTCATTTGCAAAATAACTCCTATCTCCTTGGGTTGTGATAAGGGTGATTGGATTGATACAAGTCCACAC
ATCCTTCTCTGAACTCTTTGGGAGCAGATGTGCTCCAATATCTTAGATTTTAAAAGGTAACACAGTGTATAACCCG
CGGATAACACAATACCCTCAGTGAGGTCTAAAGCAGAGCCTCTTACTTAAGCACACTAATGTTTCTCCAGTGAAA
GAAATACGCTGAGGGGGAGAAATGAAAACTTGTCATTTCAGGTCACATTTCGCTGCTCAGTGCATTTGCATACAC
AATCCTTTTCCCTAACACGCCCAACTTTAGGCTTTATGAGCATTTAGGTTTTGGAACTGGTTTAGGAATTGGATCCA
TACATTTGTAAGCATCATGTTAGTGTTCATTATGTGCAGAACTGAGTTCTTTCTATACTTGATGACCTTTGGCTTGTC
ATTATAAGAGCAGGTAAATCATTGTGGTTTAAAGTGAGTATGGTGGGGGTCATGTACTTTTATGTATTTTTCACAC
ATTACTATTTCATTTGGGTTTCGGGTCTATCCCTTTTTCCTGTTATAAACAAACTAGAAAGATTGAGGACAAGGTGT
GGCAGAAACATGCCATGTTCTTCTGAACTTCCTTTTTTTTTTTTAATTTTAAAAGAATATCTTTTTCTAGGACTTCTTT
GGCAATCGAGTGGTTAAGACGCTACTTCTACTACAGGGGAGACGCTGCTTCTACTACAGGGGACTCAGGTTTGAT

CCCTGGTCGGAGAACTGGAGTCCTTAAGCCACGGGGAGAGGCCAGAAACAAAACCGTTTCTTTCAAAGATTTAA
AATTTCTGAATTTTAAATCACAAAACTGTGATTTCTGCTTTCATTTGCCTCTCCTCTGTAGTGCACATTAAGAACAG
AAAACTGCCCAGTAAATCTTAATCTTTTGATCTGCTTTGCTGCCAGCGAATCATGGCTAGAATATCTGGTGTAGAA
AATTTTTTAATTTTAAGTAAATTAAGTGCACTTCAGACTTTAGTTTCCTTTTCCAAAGAGAGGAGCCTCCTGAGCTT
AGGACTGTGGGGTTAGCTGTCTTATATAAACTCATGGTTAGTATTACCATTGGCCTGAATGTCTAGCAATCTGGTA
TGTTTTATTCTATTTAAAAAATTCATTTAAAATCTATTAAAATCCATTGACAAATCTCTTGAAGCTTATATTAGAAAA
TAAGTAGACCTCTAGGTTTTCATCCTCTTTTCAACTACTGCGTGTATTTGCTAGTCAGAACCTGACCAAAGGACAA
TGTGAAATGCTTGAAAAAAATAGTGTATGCTTTTGAAAAGATACTATAAGCAGAAACTGTGTACTTTGAGGGGCAA
ATTTAAAAATTTAAAGGAAATGTTAGTATCCTTAAGTCTGTAACAAACATCGATCGACAGAAGTAATTAAATACAT
AGGTATTTAAGTACTACAGGAGAATAATCTTTTAGATTAATTTGCTGGCATATATCTGTTTTCTGAAGAACTTCCAA
TTCCACTTTTACTCATTTATAAATCAGTAGTCTATTAGTTCCTAAAAATAATTCTCTATAGACGCAAAGCTGATACT
GGGGTTTTTAGATTCGTGTTCTCTAACATTTCTTTATCACTTATCTTTAGTCTGCCTATAAATATATTTGTGTGAGGA
CTTTGATTCCATTGCCTCGTTAAGGCATACCAATGTGGCCCACTGAAAATAACCTGGAGGGGCTTCCTAGGTGGT
CTAGTAGCTAAGACTCTGTGCTGCCAGTGCAAGGGGCCCAGGTTTGATCCCTGGTCAGGGAACTAGATCCTGCTT
GCTGCACCTAAGGTTTTGCATGCCAAAACTAAGACCTGGCACAGCCACACACACACACACAAAAGAATAACAAA
AAAACATGACTTGGAAGGTTAGATCAAAATTGCCGGAAATCATCCTGAAGTGAGCCTTGTTTCTTTGTATAAAGTT
GCCTTGGTTGCTGCTTTGCATCATACTGATGTGGGGTATGTATTTTAAAGATGATTTTATAGTTTAGTATAGTTTTTG
TGTCTAGTGATGGCTCCAAGTTCACTTTTCTCTTTAGTGTGGTTAATCTGTGGGGTACATTAGGTCACTTGGTACAT
TTTATTTTTAGTAGTCTCACAGTATTTTAAATCTTAACATTTTACTCCTTTCCCAAGTAAGGAACCAAGGCCAGCAA
TCGCTCATGGTGAGGCTGCTCCGTTTTTCAACCTCGCTGCTCAAAACCCAAGGAGCTTAGGAAAGAAACCAAGAT
GTCTTGGGTTCTTCTGCTTTTGTTTTCTATTTTAAGGTTTAACACAGGTAGATTAACTTTTAGTTTTTAGTTTAATAAA
CTTGTTTTAAAAGTGAGATTTATAGGATTTGAGTGTATGTGTGTGCATGCATGCAAGCCTGCAGTAGGACATTGAC
TGCAGCAATGGAAAACAGGCCTGGGGCTTAAACATAGCAGATGTTTATTTCTCACATGAAAGTCTGAAAAGTGGT
CCGTGATCTGCCTGTGTGCTTGGTCCACATTCATCGGGGACCGTATTTTGTTGCGCTGTCATCTTCAACACGTAGC
TTTTTTTTTATCTTATGGTCTAAGATGACTACTCCTTTCACCATCATATATATCTTCTGTGTAGCAGAAAAGAGGGA
AGAGATATGGTGCCTTTATGGGACAACTCAGAACTTGCAGGCAGTTTAGACCTTACGAGTATAACCTTAACGAGC
AAGGGAGTTTGGCAAATCATGTACCCAACTAAATTTTCTCAACGTTGGTTACTCATCAGTATTTACCAAAAGAAAA
AAAGACCCACCAGTGTCATACAGAGGTCCATCTAGTCAAAGCTATGGTTTTTCCAGTAGTCCTGTATGGATGTGA
GAATTAGACTATAAAGAAACCTGGGCACCAAACAATTGATGCTTTTGAACTGTGGCGTTGGAAAAGACTCTTGAG
AGGCCCTTGGACTGCAAGGAGATCAAACCAGTCAATTCTAAAGGAAATCAGTCCTGAATATTCATTGGAAGGAC
TGAAGCTGAAGCTCCAATACTTTGGCTACCTGATGTGAAGGACTGACTCACTGGAAAAGACCCTGATGCTGGGC
AAGATTGAAGGCAGGAGGAGAAGGGAACGACATAGGATGAGACGGTTGGATGGCATCACTGACTTGATGAACA
TGAGTTTGAGCAAGCTCATGGACAGGGAAGCCTGGCATACTGCAGTTCATGGGGTCGCAAAGAGTCGGACACA
ACTGAGCGACTGAACTGACTGACTGTGTTATATAGCCGTTGGGATTTATACATCACCAGTCTTGGATAACCCTGG
AGAATATTTCCTTACAGATCTGTCATGTATAGAGAAGCATGCATGTGTGTTTGCTCACTCATGTCTGAATCTTTGTG
ACCCCACAGACTGTAGTCCACCGGGCTTTTCTGTCCATGGGATTTCCCAAGTAAGAATAACTGGAGTGGGGTGCC
GTTTCCTTCTCCAAGGGACCTTCCCGACCCAGGGACTGAACACACATTTCTTGCATTGCAGGTGGGTTTTTTCACT
GCTGAGCCATCGGGGAAATGCTACATGCAGAGAAGGATCTCCTAATTCTTAATTTAGGAAAGGCTTTCTAAGGAG
AAAATTGTCACCAAAAAAATAAAAGTATGGTTTAAAAAAATAAAATCAATAGAGGTTAAAAAGTAAACGTTTTTC
ATCTAATTTGCTTTGAAATAGTTTTAGAGCTTCACTATGTCGTACAAAATTGCACCCTCCTCATGTTTTTTGAGTCTT
CAGCATCTTTCTGTATCAGCAATATAGCTCTGTCTTCACTTGACAAAATGTTATTTAGCAATTATTTGGGGATTTTTT
TAATTAAAATTGGAGTATAGTTGATGTACATCATTGTGTTAAGTTCAGGTGTTCAGTGAAGTGATTCAGTTATATAT
AGATCATTTTTCAGATTCTTTTCCATTATGTTTTTTTACAAGATGCTGAATATAGTTCCCTGTGCTACACAGCAGGTC
CTTGTTTATTTTATATACAGTAGTGTGTATCTGTTAATCTATTTGGGGGATTTTTAAACAGTGCTGTTAATGTAGCTG

TCACATACTTCATTTGTGATTACATTTTTTTTTGTTAGTTGATGAATTATAGATTGCTCCCTGATTAAAACAGGTTTA
TAAAGCTTCTTAGTTCATTTTGTTCTTGCCTCAGAAATGTATAGCTTGGTCTTGCAGTGTTCTGAATTTTCTCTGAGA
GTACATGTCATGTTGAAAGTCGGAGGCTGTTGGCGTGAAAAGCAGACAAACAAAAACTGAGATGGTTGCCCACT
GTTAAATTTAGAATGAGGGACTTCCCTGGTGGTCCAGTGGATAAGACTTCATCTACCAATGCAGGGGGTGCAGGT
TCAGTCCCCGGTTGGGGAGCTAAGATTCCACATGCCCTGTGGCTAAAAAAAACCAGAACATAAACAACGGAAGT
CTTGTAACAAATTCAGTAAAGACTTTTTTTTTAATAATGTGAACCATTTTTAAACAATTCAGGGTGAAACATTAAA
AAATAGATCTGGTTTTAAATTCTGGATCAATTGTGCGTAAGTCCTTTCTGTTGAATATTAGAGTTTTAACAGATTGT
ACGTTAGTAAAGCATTAGAAGAATTGGTATTTTTCACATGTGATTTGAATGCTGTAACATTAAGGATGTATGAAGT
TCTTTGCATGTTTTTTAAAAAAATGTATAGCGTGACAATGTTGGTAGAAGATGCCAAGAGACCTGCGAGGTTGTG
CACTCCCAGTGGTTGGTCCCCATATCCTTTTTACCGTTGGCACAAGGATCCCCTCCACCACTGTTACCAATGTATA
CCTACATTTCCCGAGTGTGTTGCCATTTCCTATTCCAGGGGATCCTCCAGACCCAAGGATAGCACCCAGGTGTCC
TGCGTCTCCTCCATTGTCAGTGGATCTTTACCACTGAGCCACCTGGGATGCCCATTGTTGCTGTTAGTTGCTAAGT
CGTGTCCAACTCTCTGTGACCCCTGGACTGCAGCCTGCCAGGCTCCTCTGTCCATGGGATTCTCCAGGCAAGAAT
ACTGGAGTGGGTTGTCATTTCCTTCTCCAGGAAGCCCATACTTGGGCCTAAAAGAGATCAAGCCCTAAAACTCAC
TCTCTTATGTTTATAGCATTTGTAAACTTGGGGTGTTGGTTAAAATACTGGGAATCTCTAACATGATCCTCCTCTTC
TGATTTAGTCCTGTATTCTTATATCCAGAAGCCACTTAAGTTGTGTCCCCTTTTCCCCTCCAGCCTTTAGTTCTTCTA
GGTCACGAAGTCTGTCCTTCTCACGTACCACCACCTCTCAGGATGCCAGCCCTGGCACCTCACCTTGGACAGGG
AGAGACGGCCCCCGGCGAGATTGGCCCAAAGATACACTAGGGGGTTTCTGCCCCTCGGATAGTCTCTTAGGCGT
TTCGTCTTTCAGAGTACTTTTCCATTGTTTTAGAAACAGCTTCTTGAGTACCAGAGCCTACAGTTTTATGGGCAGAG
TTTAGTTTCAACAGCAAATCATGAAAAATTACAATTTACATTTAGTTATACTCTAAGTCAACTCGCATGTCCTGTTA
GAAGCAGGTTCCCAGCCTTTTTAAAGAACAGTTTATCTTTCTTTTAAACAAATGGAGGTATTACAAGGGACACAGA
TGGAAGACAGGTTGCCACACCAAAATAAATTAATGCAGTTTCCTTCTCCCAAAGGACATTCCTCTTAAAATGAAG
GGTGGGAGAATGGGTTAAATGTACAGAACTTTAGGTTGCTAGTTAGTCCCTTTACAGTCGAGAAGCACAGAGACT
AGCCAGCCTTCTAATAATTGTTTTAAAAGTAGATGGCTTGAAAAGCCCTGGAGGCAGAATATTTAATTGCTTGCGT
GCTAGTTCCTAAGAATTAATAATTAAGCAGTTCAGCATGAAATGGACTTGCAGTCTACCATGTAGGCTTTCTTTCA
GTCTGGTGGAAGCTCTGGATGGTTACAGCCTGATTTATTTGCTCTTATTGCTTACTCCCAGAACCAGACTTTGATG
GGGAGGTAAGACAGACCCCCAATGGAGGTCACTTTAGGGTAGGGGCTGGAAGGCTGTGAGCAGAAAGCCAAG
CCCATCCCTGTGGCTGGAACTTCACATGGTGAGCACATAGCAACTTCTGATAAACAGGCAGTTCAGGCTTTCAGT
CCCACTTCCTGCCTTGGAACTTTTGACAACTTGAATGGGAAGAAGTCATCACATAAAATGCTGCATAATATTTCCA
GAGCGGCATGACTGTGAAAAAATAACTGGCATTCTACACTCATAGACTTAGAGGACGAACTTTTGGTTGGTGGG
GAGTGTGGCGGGGGGCATGGGGGAAGGGACAGGGAGTTTAGGATCGATGTGTATACACTGCTATATTTAAAATG
GATAACCAACAAAATCCTGCTGTATAACACAAGGAACGCTGCTAAGTGTGATGTGGCAGCCTGGATGGGAGGGG
AGTTTGGGGGAGAATGGCTACATGTCTATGTATGGCTGAGTCCTTTTGCTATCTACCTGAAACTGTCACAATATTG
ATAATTTGCTATACCCCAAAAACAAAAGAAAAATTAAAAACTATATATATATAACTGGCAATCTAAAAGCTAGCA
TAGTCTCCTATTGCAGCAGGGGTAACCATGGCTATTAATTTATTGATCATTTTTTAAATTTTATCTGGTTTCCCTTTG
ACTGAAAGCAGTAGAAGTAAGATTTCACATCGGAGAAAACATTCTTTAATAATAGAGAAACTTCAGAGTAGAATT
ATTTTTCTTTAGTCTTGTGATACTCACTGATGAAAGGCAGGTGTGTTTCTTGAGCAAAAATCCTGCTACATATGTTG
AAATGAGTAGAAAACATATGATGAGCATGATGACTAGCCAGCATTTAATACTTGTTTAAAAGTGGACTGCCTTAA
AAGCGCTGGAGGTGGACTATGTGATCATTTGCCCATGCCCCAAAATGTTTCCGTCGTGTGGTCTTGGACAGGCCC
TGAGTGATACGACATAGGAGGCCTTTGGTTTTTCATAACCTGCTCGTAGCAGAAGTAGGCAGACCGACTCGTTTC
ACTGGCCTGGGCTCCTGAGATCTTTAGAATCACACCTGCCATCCCAGGACCCTATGCCTCAGAAAATTTCATTTCC
ATGCCTCTGTAAGCTAATATCTAATAAACTTCATGTATAAAATGATGTATATCGGGTATTACTGCAGCTGTTGACA
CAGGTCTGTACTCATGGGGCTTTGCTGATTTCACAGGACTGAGCTGTCTTTCATTATTTAGTGGGCATTTTCATATA
GGTCATGTGAAGGTTGTCAAGAAAGGCCTGAATGCTTTGCACAGGAACTGAGATTTTGATAGATATGGATTACCT

CCCCTTACCCCCAGTCTTTTCTTTGGAAATAACTCCCATGATTTCAGCCTCTCCAGAAGTATGAATGGGGCTAGTG
TCTTTTCTCAGGGGAAACTGTCATTTCGCTGACGTTTCCCAGAAGGAAATAGTGATGGACATACTGATAAGAAAG
GAGGACAAGGGAGAAGCTGGAGAGTTTTGGGTGGAGTTGGGGGGTGATTTTGTTTAGGGGGACATTTAACCACT
TTTTTTCTGTTTTGTAGTTTCATTTACTTATTTTTGGCTGTGCTGGGTCTTTGTTGCTGTGCAAGCTCTTCGCTAGTTG
CGGCGATTGGGGCTACGCTGGTTGCAGTGCACAGACTTCTGACTGCAGGAGGCTTCTCTTGCTGCAGAGCGCGG
GCTCTTGGCCACGCAGGCTTCAGTAGCTGCGGCTCCCAGGCCCTAGAGCACAACCTTAGTAGTTGTGGCCCACA
GACTTGGTTGGTCTGTGTCATGTGGGATCTTCCTGGATCAGGGATTGAACCCATGTCTTCTGCACTGGCAGGCTG
AT
